# Trends in loneliness in 17 European countries between 2006 and 2015: A secondary analysis of data from the European Social Survey

**DOI:** 10.1177/13591053241278473

**Published:** 2024-09-18

**Authors:** Simone Amendola, Agnes von Wyl

**Affiliations:** Department of Applied Psychology, Zurich University of Applied Sciences, Zurich, Switzerland

**Keywords:** cross-national, disconnection, Europe, loneliness, trends

## Abstract

The present study analysed changes in loneliness between 2006 and 2015 and associated factors using publicly available data (*N* = 128,718) from the European Social Survey from 17 countries. The study protocol was pre-registered on the Open Science Framework (https://osf.io/eq63j/). Loneliness-weighted prevalence (and mean) decreased from 30% to 27% over time. The decreasing trend was significant for both sexes. Young and old age groups reported a decline in loneliness over time while other age groups did not. Loneliness did not demonstrate a significant decline – but rather a stable trend – in persons with disability and first- and second-generation immigrants. Sociodemographic characteristics, social factors, well-being and psychological distress were associated with loneliness. These findings update those from previous studies indicating that loneliness trends and differences between European regions might be better explained by differences in psychological distress.

## Introduction

### Background

The COVID-19 pandemic and national social distancing measures to contain the spread of the infection gave new impetus to the study of loneliness and social isolation in recent years. Loneliness may be defined as ‘a response to a discrepancy between desired and achieved levels of social contact’ (p. 8; Peplau and Perlman, 1982) related to unmet social needs (e.g. attachment, social integration, care and nurturing) (Killeen, 1998; Russell et al., 1984; Weiss, 1973). It indicates the experience of feelings of isolation, disconnectedness and not belonging (Holt-Lunstad et al., 2015). Loneliness is different from social isolation, defined by the number of persons in the environment (Jeste et al., 2020). According to Wang et al. (2017) social isolation indicates ‘both objective social contact and subjective perceived adequacy of contact’ (p. 1453), that is, inadequate quality and quantity of social relations.

Jeste et al. (2020) authored an important viewpoint about ‘the Modern Behavioral Epidemic of Loneliness’ in the influential journal *JAMA*. The authors discussed how loneliness might represent a key risk factor explaining the increasing rates of mortality from suicides and opioid overdose over the last two decades. Social disconnection would be a result of globalization and the rapid growth of technology use and social media (Jeste et al., 2020). Concurrently, another prominent journal, *World Psychiatry*, published a relevant commentary by Green et al. (2020) reflecting on the opportunity to apply concepts from schizophrenia research to the study of social disconnection in the general community population due to alarming rates of social isolation. Despite this, the authors warned against the medicalization of social disconnection (‘social disconnection by itself is not a clinical disorder’ p. 177). Moreover, other scholars debated the possible recent increase in the rate of severe social isolation (Amendola et al., 2023) and a pathological or prolonged form of social withdrawal and isolation called *hikikomori* (Kato et al., 2020; Rooksby et al., 2020; Roza et al., 2021; Wong, 2020).

Loneliness has important public health implications since it influences both physical and mental health. A meta-analysis of 70 longitudinal studies (*N* = 48,673) demonstrated that loneliness, social isolation and living alone increased the likelihood of mortality by 30% (Holt-Lunstad et al., 2015). The result was consistent across gender, length of follow-up and world region. Initial health status and participant age influenced the findings, that is, social disconnection was more predictive of death in samples with poor health status and mean age lower than 65. Furthermore, a recent comprehensive meta-analysis of 144 studies (*N* = 18,512) revealed that loneliness had medium to large effects on depression, anxiety, suicidality, overall health, quality of life, life satisfaction, functional disability, sleep and cognition (Park et al., 2020).

Considering these findings, it is of foremost importance to examine whether and how loneliness changes over time, whether specific groups are particularly at risk as well as factors associated with change.

### Trends in loneliness over time

Previous studies on loneliness trends have mainly been conducted with samples of older adults. Honigh-de Vlaming et al. (2014) analysed loneliness scores in a sample of Dutch elderly people aged 65 years and above between 2005 and 2010. They found no difference in loneliness mean scores over time. This result was confirmed by Dahlberg et al. (2018) studying trends in loneliness between 1992 and 2014 in Swedish older adults.

However, participants with activity limitations reported an increase in loneliness (Honigh-de Vlaming et al., 2014). According to the authors, persons with activity limitations may have more difficulty engaging in society in recent years especially due to the disappearance of physical resources from the neighbourhood, such as small shops and service points (Honigh-de Vlaming et al., 2014). Further, loneliness was positively associated with being male, age, not being married, income problems and chronic diseases. Dahlberg et al. (2018) confirmed the association between loneliness and not being married, psychological distress, lack of social support and fewer social contacts.

In contrast, Aunsmo et al. (2023) showed that the prevalence of loneliness decreased between 1984 and 2019 in Norwegian adults aged 70 years and older. Subgroup analyses pointed out that the prevalence of loneliness among the oldest adults living alone increased from 2006 to 2019. Furthermore, loneliness was significantly more common among women, the oldest and those living alone. Tesch-Römer et al. (2013) also found an overall slight decrease in loneliness scores between 1996 and 2008 in a representative German sample aged 40–85 years. The authors showed trend differences according to age for which middle adults reported an increase in loneliness between 2002 and 2008 while older adults a decline. They hypothesized that the increased risk for loneliness in middle adulthood could be due to the increased fragility of social networks (Tesch-Römer et al., 2013). Furthermore, men showed higher loneliness compared to women and this difference was stable over time.

Considering adulthood overall (18–74 years of age), Blom et al. (2020) established that loneliness did not show significant change over time in Swedish adults between 2000 and 2016. While considering subgroup analyses, loneliness increased in women and the young age group (18–34 years).

Only a few studies examined loneliness trends in young people. Clark et al. (2015) found declines in loneliness score between 1978 and 2009 and between 1991 and 2012 in US high school and college students, respectively. In addition, White students reported lower loneliness than Black students, Hispanic students or students of other races. According to the authors, cultural changes such as increases in extraversion and self-esteem as well as society acceptance and economic opportunities – rather than the use of social media – could explain the decline in loneliness over time (Clark et al., 2015). On the contrary, Twenge et al. (2021) showed a stable trend in loneliness between 2000 and 2012 whereas loneliness increased thereafter until 2018 using data from 37 countries. The authors stressed that the rise of smartphone access and internet use may be related to the positive trend in loneliness.

### The study aims and hypotheses

In light of the above, the first aim of the present study was to analyse changes in loneliness between 2006 and 2015 overall and according to sociodemographic characteristics using data from all four rounds of the European Social Survey (ESS) including data on loneliness. In light of the findings discussed above, the related hypothesis was that loneliness will increase in young adults, remain stable in middle adults and decline in older adults. Increases in some groups were expected such as persons with activity limitations (Honigh-de Vlaming et al., 2014) and those living alone (Aunsmo et al., 2023). Further, loneliness trends were analysed according to sociodemographic characteristics such as living area, country, European region, education and immigration status, to expand available evidence (d’Hombres et al., 2021). The secondary aim was to explore the relationship between loneliness and social factors (i.e. victimization, emotional support, social trust, meetings and activities), lifestyle behaviours (i.e. physical activity, cigarettes and alcohol use, binge drinking and body mass index), distress and well-being (i.e. happiness, general health, enjoying life, symptoms of depression, feeling everything like effort, restless sleep, sadness, lethargy and lack motivation) to better understand factors accounting for the trend in loneliness, if any, as encouraged by previous research (e.g. Aunsmo et al., 2023). Overall, it was hypothesized that loneliness will be positively associated with symptoms of psychological distress and poor lifestyle behaviours, and negatively with measures of well-being (Dahlberg, 2022; Dahlberg et al., 2018; Holt-Lunstad et al., 2015; Park et al., 2020; Tesch-Römer et al., 2013), despite the analysis of some factors (e.g. victimization) was exploratory in nature. The unique contribution of the present study over previous research was thus to conduct a comprehensive investigation of loneliness trends in Europe, increase results generalizability going beyond country-specific analysis, and provide further insights into potential determinants of loneliness in addition to sociodemographic characteristics (d’Hombres et al., 2021).

## Methods

### Data

Our analysis is based on publicly available data from the ESS rounds 3, 5, 6 and 7 (European Social Survey European Research Infrastructure [ESS ERIC], 2018a, 2018b, 2018c, 2018d). The ESS is conducted in several European countries using strict random probability sampling, a minimum target response rate of 70% and rigorous translation protocols. The face-to-face interview is conducted with persons aged 15 and over, residents within private households, and includes questions exploring a variety of social indicators. Data collection occurred between August 2006 and September 2007 for ESS round 3, between August 2010 and January 2012 for ESS round 5, between August 2012 and December 2013 for ESS round 6 and between August 2014 and December 2015 for ESS round 7. The dataset (*N* = 129,217) including data from 17 countries with available data from ESS rounds 3, 5, 6 and 7 was downloaded from https://ess-search.nsd.no/CDW/RoundCountry on July 10, 2023. Approval from an ethics committee was not requested because we conducted a secondary analysis of publicly available anonymized data. The study protocol was pre-registered on the Open Science Framework (https://osf.io/eq63j/).

### Measures

#### Loneliness

With regard to loneliness in the ESS, the interviewer reads out the following statement to the respondent: ‘Using this card, please tell me how much of the time during the past week you felt lonely?’. Possible answers are 1 = ‘None or almost none of the time’, 2 = ‘Some of the time’, 3 = ‘Most of the time’, 4 = ‘All or almost all of the time’ and 8 = ‘Don’t know’. Previous research demonstrated that loneliness single-item scores were highly correlated with scores of widely used composite measures of loneliness (Grygiel et al., 2019; Mund et al., 2023).

#### Sociodemographic characteristics

All variables used in this study are described in Appendix. Sociodemographic characteristics explored were sex, age, number of people living regularly as members of the household, living alone, living area (e.g. big city, small city, village), country and region of Europe according to EuroVoc divided in Northern (Denmark, Estonia, Finland, Norway, Sweden), Southern (Portugal, Spain), Western (Belgium, France, Germany, Ireland, Netherlands, Switzerland, United Kingdom), Central and Eastern Europe (Hungary, Poland, Slovenia), level of education, being permanently sick or disabled, immigration status (second generation, first generation), being in employment/education, religiosity and severe social isolation. As done for a previous study (Amendola et al., 2023) examining severe social isolation as a proxy for a condition of high risk for hikikomori, the presence of all the following indicators was considered to identify persons in severe social isolation: (1) social meeting with friends, relatives or colleagues less than once a month or never, (2) taking part in social activities less than most or much less than most compared to others of same age, (3) not working (or not away from work temporarily) during the last week, (4) not actively looking for a job during the last week and (5) not being in education (not paid for by employer), even if on vacation, during the last week.

#### Social factors

Social involvement was measured by the frequency of social meetings and of taking part in social activities compared to others of the same age.

Emotional support was investigated by asking respondents the number of people with whom he/she can discuss intimate and personal matters and was recoded as ‘none’ or ‘at least one’.

Social trust was measured using the Social Trust Scale (Breyer, 2015). Three items explore the respondent’s trust in other people, his/her belief that most people would try to take advantage of him/her, and the belief that most of the time people try to be helpful or that they are mostly looking out for themselves. Each item is rated on a 11-point Likert scale, with higher scores indicating high social trust. A total mean score is computed by summing items responses and dividing by the number of items answered.

Finally, one item examined respondent or household member victimization due to burglary/assault during the last 5 years.

#### Well-being and psychological distress

Respondents’ well-being was explored by considering happiness, subjective general health and enjoying life. Indicators of psychological distress were single items on depression, feeling that everything is an effort, restless sleep, sadness, lethargy and lack of motivation during the last week.

#### Lifestyle behaviours

Regarding lifestyle behaviours, engagement in sports or other physical activity, number of cigarettes smoked on a typical day, frequency of alcohol use and binge drinking and body mass index were considered for analysis.

### Statistical analysis

Data were initially inspected to ensure the availability of information – across different ESS rounds – on the variables of interest. Data from participants who responded with ‘don’t know’, or did not provide an answer to the statement exploring loneliness (*n* = 499, 0.39% of the sample), were not included in study analyses because did not provide useful information on the experience of loneliness and were, thus, handled using listwise deletion. Therefore, the sample of valid responses for statistical analyses was *N* = 128,718.

We used a complex sampling design to obtain weighted prevalence/mean and results. Specifically, clustering, stratification and weighting were considered. The analysis weight variable is suitable for all analyses, such as comparing multiple countries or studying multiple countries as a group (Kaminska, 2020).

Descriptive statistics (mean, prevalence and standard error) were used to explore the characteristics of the sample. Trends of loneliness item mean score over time were analysed overall as well as by the sociodemographic variables: sex, age group, European region, country, city, living alone versus living with other(s), presence of disability, immigrant status (first generation, second generation, citizen), employment/education versus not being in employment/education, level of education (low, intermediate, high), religiosity and severe social isolation.

The association between loneliness and the variables of interest was tested using adjusted multiple linear regression models including time (i.e. ESS round) and the sociodemographic variables (except severe social isolation due to overlap with other variables, such as employment/education and social involvement), social factors and psychological distress and well-being or lifestyle behaviours (excluding time because data on lifestyle behaviours are available for ESS round 7 only). As part of sensitivity analysis, the above analysis was repeated using ordered logistic regression with loneliness as the ordinal dependent variable.

All analyses were performed in RStudio using the ‘survey’ package (Lumley, 2020).

## Results

### Trend in loneliness

Table 1 shows loneliness weighted mean and results of test for linear trend according to sociodemographic characteristics. Loneliness weighted mean was 1.38 overall (Table S1 in Supplementary material) and decreased from 1.40 in ESS round 3 to 1.36 in ESS round 7 (Table 1). Weighted descriptive characteristics of the sample are reported in Table S1 while Figure S1 displays loneliness weighted mean trends by sociodemographic characteristics.

**Table 1.** Loneliness weighted mean (and standard error) according to round 3, 5, 6 and 7 of the European Social Survey (ESS) and subgroup with the test for linear trend.

Subgroup	ESS 3 (2006–2007)	ESS 5 (2010–2012)	ESS 6 (2012–2013)	ESS 7 (2014–2015)	Estimate (SE)	*t*	*p*
Total sample	1.40 (0.01)	1.36 (0.01)	1.38 (0.01)	1.36 (0.01)	−0.009 (0.002)	4.214	0.001
Sex							
Male	1.33 (0.01)	1.32 (0.01)	1.32 (0.01)	1.31 (0.01)	−0.006 (0.003)	−2.106	0.035
Female	1.46 (0.01)	1.41 (0.01)	1.44 (0.01)	1.41 (0.01)	−0.011 (0.003)	−3.717	0.001
Age group							
15–29 years	1.39 (0.01)	1.34 (0.01)	1.36 (0.01)	1.35 (0.01)	−0.010 (0.004)	−2.248	0.025
30–49 years	1.35 (0.01)	1.34 (0.01)	1.35 (0.01)	1.32 (0.01)	−0.006 (0.003)	−1.786	0.074
50–64 years	1.39 (0.01)	1.35 (0.01)	1.38 (0.01)	1.36 (0.01)	−0.005 (0.004)	−1.238	0.216
65–79 years	1.47 (0.02)	1.38 (0.02)	1.41 (0.01)	1.37 (0.01)	−0.022 (0.005)	−4.12	<0.001
>79 years	1.68 (0.04)	1.70 (0.04)	1.65 (0.03)	1.63 (0.03)	−0.015 (0.012)	−1.207	0.228
European region							
Northern	1.29 (0.01)	1.27 (0.01)	1.28 (0.01)	1.29 (0.01)	0.001 (0.003)	0.302	0.763
Southern	1.44 (0.01)	1.42 (0.01)	1.42 (0.01)	1.43 (0.01)	−0.003 (0.005)	−0.708	0.479
Western	1.38 (0.01)	1.34 (0.01)	1.37 (0.01)	1.34 (0.01)	−0.010 (0.003)	−3.704	<0.001
Central and Eastern	1.49 (0.02)	1.44 (0.01)	1.46 (0.01)	1.43 (0.02)	−0.014 (0.005)	−2.558	0.011
Country							
Belgium	1.40 (0.01)	1.34 (0.01)	1.42 (0.01)	1.36 (0.01)	−0.004 (0.006)	−0.725	0.468
Switzerland	1.24 (0.01)	1.24 (0.01)	1.28 (0.02)	1.26 (0.01)	−0.006 (0.005)	1.347	0.178
Germany	1.37 (0.01)	1.30 (0.01)	1.31 (0.01)	1.27 (0.01)	−0.022 (0.005)	−4.824	<0.001
Denmark	1.21 (0.02)	1.21 (0.01)	1.22 (0.01)	1.23 (0.02)	0.005 (0.005)	0.866	0.386
Estonia	1.49 (0.02)	1.46 (0.02)	1.47 (0.01)	1.42 (0.01)	−0.014 (0.006)	−2.321	0.020
Spain	1.41 (0.02)	1.42 (0.02)	1.42 (0.02)	1.42 (0.02)	0.004 (0.006)	0.658	0.51
Finland	1.27 (0.01)	1.26 (0.01)	1.28 (0.01)	1.24 (0.01)	−0.006 (0.004)	−1.414	0.157
France	1.48 (0.02)	1.45 (0.02)	1.50 (0.02)	1.47 (0.02)	0.001 (0.007)	0.108	0.914
United Kingdom	1.36 (0.02)	1.36 (0.02)	1.33 (0.01)	1.32 (0.01)	−0.009 (0.005)	−1.83	0.067
Hungary	1.60 (0.03)	1.52 (0.02)	1.63 (0.02)	1.52 (0.02)	−0.011 (0.008)	−1.413	0.158
Ireland	1.35 (0.02)	1.40 (0.02)	1.32 (0.01)	1.34 (0.01)	−0.004 (0.005)	−0.844	0.399
Netherlands	1.28 (0.01)	1.18 (0.01)	1.27 (0.01)	1.22 (0.01)	−0.011 (0.004)	−2.471	0.014
Norway	1.26 (0.01)	1.24 (0.01)	1.22 (0.01)	1.27 (0.02)	−0.000 (0.005)	−0.044	0.965
Poland	1.47 (0.02)	1.42 (0.02)	1.43 (0.02)	1.41 (0.02)	−0.012 (0.007)	−1.811	0.070
Portugal	1.58 (0.02)	1.43 (0.02)	1.37 (0.02)	1.50 (0.03)	−0.034 (0.007)	−4.757	<0.001
Sweden	1.32 (0.01)	1.31 (0.02)	1.32 (0.02)	1.35 (0.02)	0.007 (0.005)	1.312	0.19
Slovenia	1.43 (0.02)	1.45 (0.02)	1.28 (0.02)	1.32 (0.02)	−0.034 (0.006)	−5.689	<0.001
Living in a city							
No	1.38 (0.01)	1.34 (0.01)	1.35 (0.01)	1.35 (0.01)	−0.008 (0.003)	−2.561	0.010
Yes	1.41 (0.01)	1.37 (0.01)	1.39 (0.01)	1.37 (0.01)	−0.008 (0.003)	−3.219	0.001
Living alone							
No	1.32 (0.01)	1.29 (0.01)	1.31 (0.01)	1.29 (0.01)	−0.007 (0.002)	−3.447	<0.001
Yes	1.83 (0.02)	1.79 (0.02)	1.79 (0.02)	1.75 (0.02)	−0.018 (0.006)	−2.984	0.003
Disability							
No	1.39 (0.01)	1.35 (0.01)	1.37 (0.01)	1.35 (0.01)	−0.008 (0.002)	−4.196	<0.001
Yes	1.79 (0.05)	1.69 (0.04)	1.73 (0.04)	1.74 (0.05)	−0.012 (0.015)	−0.752	0.452
Immigration status							
No immigrant background	1.39 (0.01)	1.35 (0.01)	1.37 (0.01)	1.35 (0.01)	−0.009 (0.002)	−4.422	<0.001
Second generation	1.44 (0.05)	1.36 (0.04)	1.41 (0.04)	1.40 (0.04)	−0.008 (0.015)	−0.534	0.594
First generation	1.48 (0.03)	1.46 (0.02)	1.50 (0.02)	1.43 (0.02)	−0.007 (0.008)	−0.833	0.405
In employment/education							
Yes	1.33 (0.01)	1.29 (0.01)	1.31 (0.01)	1.29 (0.01)	−0.009 (0.002)	−3.752	<0.001
No	1.52 (0.01)	1.48 (0.01)	1.49 (0.01)	1.48 (0.01)	−0.009 (0.004)	−2.508	0.012
Level of education							
Low	1.51 (0.01)	1.44 (0.01)	1.48 (0.01)	1.48 (0.01)	−0.006 (0.005)	−1.28	0.201
Intermediate	1.37 (0.01)	1.33 (0.01)	1.34 (0.01)	1.32 (0.01)	−0.012 (0.003)	−4.384	<0.001
High	1.28 (0.01)	1.29 (0.01)	1.29 (0.01)	1.26 (0.01)	−0.006 (0.004)	−1.546	0.122
Religious							
Yes	1.40 (0.01)	1.36 (0.01)	1.38 (0.01)	1.37 (0.01)	−0.007 (0.003)	−2.74	0.006
No	1.39 (0.01)	1.37 (0.01)	1.38 (0.01)	1.34 (0.01)	−0.010 (0.003)	−3.194	0.001
Severe social isolation							
Absent	1.38 (0.01)	1.35 (0.01)	1.36 (0.01)	1.35 (0.01)	−0.007 (0.002)	−3.392	<0.001
Present	1.92 (0.04)	1.84 (0.05)	1.84 (0.01)	1.76 (0.01)	−0.036 (0.015)	−2.449	0.014

SE: standard error.

Regarding loneliness weighted prevalence regardless of ESS round, 28% of the sample reported some degree of loneliness (some, most, all or almost all of the time) during the previous week (Table S1). Loneliness decreased from 30% to 27% over time (Table S2). Figure S2 shows loneliness category weighted prevalence trends by sociodemographic characteristics.

ESS round and loneliness were negatively associated, that is, loneliness decreased over time (across ESS rounds). The decreasing trend was significant for both sexes. Young and old age groups reported a decline in loneliness over time while other age groups did not. Loneliness declined both in Western and Central and Eastern Europe while the trend was stable both in Northern and Southern Europe. Regarding the country level, a significant decline in loneliness was observed in five (i.e. Slovenia, Germany, Portugal, Netherlands and Estonia) of the 17 European countries analysed while no significant trend was found in the others. Furthermore, loneliness decreased over time irrespective of living conditions (alone vs with others, in a city or not), severe social isolation and religiosity. To note, loneliness decreased in persons without disability whereas it showed no trend (i.e. was stable) in persons with disability. Similarly, it did not demonstrate a significant decline – but rather a stable trend – in first- and second-generation immigrants. Regarding education level, loneliness declined in persons with intermediate education while it was stable in low and high-education groups.

Overall, the results of the regression models reported above did not change when loneliness was analysed as an ordinal dependent variable using ordered logistic regression as part of sensitivity analysis (results not reported in the text).

Table S3 shows associations between loneliness and the variable of interest controlling for the ESS round. Results of the multivariable regression model predicting loneliness by sociodemographic characteristics are displayed in Table 2. All sociodemographic variables were significantly associated with loneliness (except for the oldest age group which was not less/more likely to report loneliness compared to the youngest one). McFadden’s pseudo-*R*^2^ of the model was 0.10.

**Table 2.** Results of multivariable regression model predicting loneliness by sociodemographic characteristics.

Independent variable	Estimate (SE)	*t*	*p*
ESS round 3	Reference		
ESS round 5	−0.044 (0.009)	−5.076	<0.001
ESS round 6	−0.024 (0.009)	−2.709	0.007
ESS round 7	−0.04 (0.009)	−4.482	<0.001
Female	0.086 (0.006)	14.528	<0.001
15–29 years	Reference		
30–49 years	−0.02 (0.008)	−2.357	0.018
50–64 years	−0.049 (0.009)	−5.388	<0.001
65–79 years	−0.127 (0.012)	−10.324	<0.001
>79 years	0 (0.02)	−0.003	0.998
Northern Europe	Reference		
Southern Europe	0.172 (0.009)	20.012	<0.001
Western Europe	0.082 (0.006)	14.692	<0.001
Central and Eastern Europe	0.23 (0.009)	26.534	<0.001
Living in a city	0.015 (0.006)	2.447	0.014
Living alone	0.482 (0.01)	48.779	<0.001
Presence of disability	0.248 (0.023)	10.934	<0.001
Citizen	Reference		
Second generation immigrant	0.077 (0.021)	3.606	<0.001
First generation immigrant	0.141 (0.012)	11.567	<0.001
Not in employment/education	0.124 (0.009)	13.979	<0.001
Low education	Reference		
Intermediate education	−0.086 (0.008)	−11.365	<0.001
High education	−0.144 (0.009)	−16.796	<0.001
Not religious	0.039 (0.006)	6.258	<0.001

*N* = 116,052.

SE: standard error.

The results of the multivariable regression model did not change (except for the significant negative association between the oldest age group and loneliness) when loneliness was analysed as an ordinal dependent variable using ordered logistic regression as part of sensitivity analysis (results not reported in the text).

### Loneliness and social factors

Social factors were all associated with loneliness (Table 3). Victimization was positively associated with loneliness while emotional support, social trust and social involvement (i.e. meetings and activities) were negatively associated with it. McFadden’s pseudo-*R*^2^ of the model was 0.14. In this model, the effects of the European region and ESS round (except the comparison between rounds 3 and 6) were still significant.

The results of the multivariable regression model did not change when loneliness was analysed as an ordinal dependent variable using ordered logistic regression as part of sensitivity analysis (results not reported in the text).

**Table 3.** Results from three multivariable regression models predicting loneliness by social factors, well-being and psychological distress and lifestyle behaviours, respectively.

Independent variable	Estimate (SE)	*t*	*p*	Standardized estimate
Social factors^a^				
Victimization	0.069 (0.008)	8.329	<0.001	0.07
Emotional support	−0.253 (0.018)	−13.888	<0.001	−0.25
Social trust	−0.035 (0.002)	−17.548	<0.001	−0.06
Social meetings	−0.029 (0.002)	−11.584	<0.001	−0.04
Social activities	−0.063 (0.004)	−16.919	<0.001	−0.06
Well-being and psychological distress^b^				
Happiness	−0.038 (0.002)	−15.505	<0.001	−0.07
General health	−0.015 (0.005)	−3.256	0.001	−0.01
Enjoying life	−0.043 (0.005)	−9.003	<0.001	−0.04
Symptoms of depression	0.097 (0.008)	12.423	<0.001	0.07
Feeling everything like effort	0.015 (0.006)	2.689	0.007	0.01
Restless sleep	0.042 (0.005)	9.093	<0.001	0.04
Sadness	0.276 (0.008)	35.531	<0.001	0.19
Lethargy and lack motivation	0.057 (0.006)	8.751	<0.001	0.04
Lifestyle behaviours^c^				
Physical activity	−0.006 (0.002)	−2.485	0.013	−0.02
Cigarettes smoked daily	0.004 (0.001)	3.854	<0.001	0.03
Alcohol use	0.004 (0.005)	0.743	0.457	0.01
Binge drinking	−0.012 (0.006)	−1.876	0.061	−0.01
Body mass index	0.002 (0.002)	1.503	0.133	0.01

SE: standard error.

a Model controlled for sociodemographic characteristics (results not shown), *N* = 113,693.

b Model controlled for sociodemographic characteristics and social factors (results not shown), *N* = 82,159.

c Model controlled for sociodemographic characteristics and social factors (results not shown), *N* = 23,403.

### Loneliness, well-being and psychological distress

All well-being and psychological distress independent variables were significantly associated with loneliness (Table 3), especially, sadness, happiness and symptoms of depression. Data from ESS round 5 was not included in this model because information on well-being and psychological distress was not collected. McFadden’s pseudo-*R*^2^ of the model was 0.32.

To note, the association between ESS round and loneliness and between European region and loneliness became non-significant in this model indicating that differences in loneliness between ESS round and European regions might be better explained by differences in well-being and psychological distress. Additional analyses (not reported in the text) found that differences between European regions disappeared with the inclusion of psychological distress (rather than well-being) variables in the model.

The results of the multivariable regression model did not change when loneliness was analysed as an ordinal dependent variable using ordered logistic regression as part of sensitivity analysis (results not reported in the text) except that (1) persons from Southern and Western Europe (but not from Central and Eastern Europe) were still more likely to report loneliness than those from Northern region, and (2) feeling everything was an effort was not significantly (*p* = 0.092) associated with loneliness. Further analyses (not reported in the text) showed that differences between Northern and Central and Eastern Europe disappeared when psychological distress – rather than well-being – was included in the model.

### Loneliness and lifestyle behaviours

Data from ESS round 7 only were used for this model because information on lifestyle behaviours was not collected in ESS rounds 3, 5 and 6. Physical activity was negatively associated with loneliness, while cigarettes smoked daily were positively associated with it (Table 3). Alcohol use and body mass index were not associated with loneliness after accounting for sociodemographic characteristics and social factors. To note, only persons from Southern (but not from Western Europe and Central and Eastern Europe) were still more likely to report loneliness compared to those from the Northern region when lifestyle behaviours variables were included in the model. McFadden’s pseudo-*R*^2^ of the model was 0.11.

The results of the multivariable regression model did not change when loneliness was analysed as an ordinal dependent variable using ordered logistic regression as part of sensitivity analysis (results not reported in the text).

Additional sensitivity analysis showed that, if well-being and psychological distress variables were also added to this model, the effect of all lifestyle behaviours variables became non-significant and significant differences in loneliness between European regions disappeared (results not shown).

## Discussion

The present study examined changes in loneliness between 2006 and 2015 using weighted data from 17 European countries. It thus constitutes a valuable analysis improving current scientific knowledge. Our findings demonstrated an overall decline in loneliness over time regardless of sex, living (in a city, alone) and employment/education conditions, religiosity and severe social isolation. Loneliness decreased from 29.88% in 2006–2007 to 26.78% in 2014–2015. Overall, the study findings do not seem to support the notion of a modern behavioural epidemic of loneliness or, better, an increasing trend in loneliness over the period analysed.

However, we found that loneliness trends varied according to age group, European country and region, disability and immigration status, and level of education. Loneliness decreased in the age groups 15–29 and 65–79 years while was mainly stable in the others. These findings supported the study hypothesis about age differences in loneliness except that an increasing trend for young people was initially expected based on findings from Twenge et al. (2021). Nonetheless, between-study heterogeneity in age (15–16 years old vs 15–29 years) and measured construct (school loneliness vs loneliness in general) might have influenced the results. According to Twenge et al. (2021) the recent increase in school loneliness could be due to the rise of smartphone access and internet use. It is intriguing to speculate whether such a result might be accurate for school loneliness only whereas the opposite – that is, smartphone access and internet use leading to a decrease in loneliness – could apply to loneliness in general as our findings imply. But, decreasing trends in loneliness were evident before the 2000s (Clark et al., 2015).

We hypothesized that some groups were at risk of an increasing trend in loneliness. Our findings partially confirmed it. Disability and immigrant background were associated with a stable – rather than declining – trend in loneliness. Some persons may thus experience more difficulty in engaging society in recent years due, for example, to the disappearance of physical resources from the neighbourhood (Honigh-de Vlaming et al., 2014). It could also be the case that digitalization and the use of new technologies have not improved opportunities for social contact and relationships for persons with disabilities and immigrants.

Regarding factors associated with loneliness, we investigated the role of sociodemographic characteristics, social and lifestyle behaviours, distress and well-being. Of relevance, the inclusion of time in the analysis enabled us to increase scientific knowledge on factors accounting for the declining trend in loneliness. When only sociodemographic variables were considered in the model, all of them were significantly associated with loneliness and the effect of time (i.e. ESS round) was significant as well (i.e. loneliness decreased over time with this effect being not explained by other variables included in the model). This indicates that individual differences in sociodemographic characteristics did not account for the decline in loneliness observed over time. Only living alone had a medium effect size on loneliness. Living condition is related to loneliness and individuals living alone might experience increased loneliness due to fewer daily face-to-face connections (d’Hombres et al., 2021). The presence of disability, living in Central and Eastern and Southern Europe, being first generation immigrant, and not working or being in education had a small effect size on loneliness. Whereas, high education and the age group 65–79 years compared to low education and age group 15–29 years, respectively, had a small effect size on loneliness. The other factors had significant but very small or negligible effect size on loneliness. Previous research has shown that sociodemographic factors and socio-cultural context may influence individual social isolation and loneliness via socioeconomic factors informing differences between European regions (Amendola et al., 2023; Kung et al., 2022; Tapia-Muñoz et al., 2022).

Social factors were all significantly associated with loneliness. However, only emotional support had a small effect size on loneliness. This result underscores the importance of having someone with whom to discuss intimate and personal matters to reduce feelings of loneliness. Other social factors (victimization, social trust and social meetings and activities) showed significant but very small or negligible effect size on loneliness. Furthermore, individual differences in social factors (and sociodemographic characteristics) did not explain the decline in loneliness observed over time.

We found that all psychological distress and well-being variables were associated with loneliness as expected. Among lifestyle behaviours, physical (in)activity and number of cigarettes smoked were associated with loneliness despite a very small or negligible effect size. Only sadness showed a small effect size on loneliness. The robust relationship between loneliness and depressive symptoms is acknowledged in the literature with reciprocal influences over time being probably the most plausible model explaining this relationship (Dahlberg, 2022). Expanding previous scientific evidence, we found that the association between loneliness and sadness is even stronger. This could be explained by pointing to poor emotional support as a common cause or shared risk factor. Accordingly, the evolutionary model of loneliness postulates that the experience of loneliness arises as a signal to promote social (re)connection and that, when this is not possible, it might increase negative affect (Cacioppo et al., 2014; Hawkley and Cacioppo, 2010). Finally, we verified that the declining trend over time and the differences between European regions (especially between Northern and Central and Eastern Europe) in loneliness were both better explained by individual differences in psychological distress and well-being. This result is in line with previous literature showing a negative, declining, trend in psychological distress throughout Europe (Beller et al., 2021).

### Limitations

The findings from the present study should be interpreted considering some limitations. First, the ESS is not aimed at studying loneliness specifically and other questions examined in this study. Consequently, available data mainly collected using single-item measures were analysed. Second, the cross-sectional design of the study limits causal inference and the results of the statistical analyses should not be interpreted as explaining causality between variables of interest and loneliness but rather their association at a specific point in time. Third, a relatively limited period was examined. Data were obtained from four observations between 2006 and 2015. It was thus possible to analyse trend linearity but for more complex analysis (e.g. changes in trend) a higher number of surveys is needed.

## Conclusions

This secondary analysis of ESS data enabled us to study loneliness in a highly representative European sample between 2006 and 2015 using weighted data. We demonstrated that loneliness showed a declining trend over the analysed period. However, some groups not registering such a decline were also found, such as persons with disability and immigrant background, middle-aged adults and those from Northern and Southern regions of Europe. To note, differences in loneliness over time and across regions disappeared when accounting for individual psychological distress. While lifestyle behaviours such as physical activity and smoking were only minimally associated with loneliness. Our findings inform public health interventions focused on preventing and reducing loneliness in the general population. Living alone, the presence of disability, poor emotional support and sadness were prominently related to loneliness. Interventions aiming at promoting social connection may thus be especially useful to improve both objective social contact and the quality of social connections (Zagic et al., 2022). Previous evidence demonstrated the efficacy of social cognitive training, social support enhancement, social skills improvement and social interaction opportunities interventions in reducing loneliness (Masi et al., 2011). Nevertheless, short- and long-term efficacy needs to be examined to prove whether such interventions have long-lasting effects (Eccles and Qualter, 2021).

## Supplemental Material

sj-docx-1-hpq-10.1177_13591053241278473 – Supplemental material for Trends in loneliness in 17 European countries between 2006 and 2015: A secondary analysis of data from the European Social Survey Supplemental material, sj-docx-1-hpq-10.1177_13591053241278473 for Trends in loneliness in 17 European countries between 2006 and 2015: A secondary analysis of data from the European Social Survey by Simone Amendola and Agnes von Wyl in Journal of Health Psychology

sj-docx-2-hpq-10.1177_13591053241278473 – Supplemental material for Trends in loneliness in 17 European countries between 2006 and 2015: A secondary analysis of data from the European Social Survey Supplemental material, sj-docx-2-hpq-10.1177_13591053241278473 for Trends in loneliness in 17 European countries between 2006 and 2015: A secondary analysis of data from the European Social Survey by Simone Amendola and Agnes von Wyl in Journal of Health Psychology

sj-docx-3-hpq-10.1177_13591053241278473 – Supplemental material for Trends in loneliness in 17 European countries between 2006 and 2015: A secondary analysis of data from the European Social Survey Supplemental material, sj-docx-3-hpq-10.1177_13591053241278473 for Trends in loneliness in 17 European countries between 2006 and 2015: A secondary analysis of data from the European Social Survey by Simone Amendola and Agnes von Wyl in Journal of Health Psychology

sj-pdf-4-hpq-10.1177_13591053241278473 – Supplemental material for Trends in loneliness in 17 European countries between 2006 and 2015: A secondary analysis of data from the European Social Survey Supplemental material, sj-pdf-4-hpq-10.1177_13591053241278473 for Trends in loneliness in 17 European countries between 2006 and 2015: A secondary analysis of data from the European Social Survey by Simone Amendola and Agnes von Wyl in Journal of Health Psychology

sj-pdf-5-hpq-10.1177_13591053241278473 – Supplemental material for Trends in loneliness in 17 European countries between 2006 and 2015: A secondary analysis of data from the European Social Survey Supplemental material, sj-pdf-5-hpq-10.1177_13591053241278473 for Trends in loneliness in 17 European countries between 2006 and 2015: A secondary analysis of data from the European Social Survey by Simone Amendola and Agnes von Wyl in Journal of Health Psychology

## Appendix

**Table A1.** List of indicators used in this study.

Acronym	Variable	Value/category
Loneliness		
fltlnl (and fltlnla)	“Using this card, please tell me how much of the time during the past week you felt lonely?”	1 = None or almost none of the time, 2 = Some of the time, 3 = Most of the time, 4 = All or almost all of the time
Sociodemographic characteristics		
gndr	Sex	1 = Male, 2 = Female
agea	Age of respondent	Ranging from 15 to 114
*hhmmb*	“Including yourself, how many people – including children – live here regularly as members of this household?”	In number
livealone	Respondent lives alone (computed based on ‘hhmmb’)	0 = No, 1 = Yes
*domicil*	“Which phrase on this card best describes the area where you live?”	1 = A big city, 2 = The suburbs or outskirts of a big city, 3 = A town or a small city, 4 = A country village, 5 = A farm or home in the countryside
city	Living in a city (computed based on ‘domicil’)	0 = No (A country village, A farm or home in the countryside), 1 = Yes (A big city, The suburbs or outskirts of a big city, A town or a small city)
*cntry*	European country	
region	Region of Europe according to EuroVoc (computed based on ‘cntry’)	1 = Northern (Denmark, Estonia, Finland, Norway, Sweden), 2 = Southern (Portugal, Spain), 3 = Western (Belgium, France, Germany, Ireland, Netherlands, Switzerland, United Kingdom), 4 = Central and Eastern Europe (Hungary, Poland, Slovenia)
eisced	“What is the highest level of education you have successfully completed?”	0 = Low (ES-ISCED I, ES-ISCED II), 1 = Intermediate (ES-ISCED IIIb, ES-ISCED IIIa, ES-ISCED IV), 2 = High (ES-ISCED V1, ES-ISCED V2)
dsbld	Permanently sick or disabled	0 = No, 1 = Yes
*brncntr*	Born in country	1 = Yes, 2 = No/other country
*facntr, mocntr*	Father/mother born in country	1 = Yes, 2 = No/other country
immigrant	Immigration status (computed based on ‘brncntr’, ‘facntr’ and ‘mocntr’)	0 = no (respondents and at least one parent born in country, or both parents born in country and missing respondent’s data), 1 = second generation (respondent born in country whereas parents not), 2 = first generation (respondent and parents born in other country)
lackempledu	In employment/education (computed based on ‘pdwrk’ and ‘edctn’)	0 = Yes, 1 = No
rlgblg	“Do you consider yourself as belonging to any particular religion or denomination?”	1 = Yes, 2 = No
hikrisk	Severe social isolation with high risk of hikikomori (computed based on the following five indicators)	0 = No, 1 = Yes
*sclmeet*	“How often do you meet socially with friends, relatives or work colleagues?”	1 = Never, 2 = Less than once a month
*sclact*	“Compared to other people of your age, how often would you say you take part in social activities?”	1 = Much less than most, 2 = Less than most
*pdwrk*	In paid work (or away temporarily) (employee, self-employed, working for your family business) during the last 7 days	Not marked
*uempla*	Unemployed, actively looking for job during the last 7 days	Not marked
*edctn*	In education, (not paid for by employer) even if on vacation during the last 7 days	Not marked
Social factors		
crmvct	“Have you or a member of your household been the victim of a burglary or assault in the last 5 years?”	0 = No, 1 = Yes
emotionalsupport (based on ‘inmdisc’ and ‘inprdsc’)	“Do you have anyone with whom you can discuss intimate and personal matters?” OR “How many people, if any, are there with whom you can discuss intimate and personal matters?”	0 = No/none to 1 = Yes/at least one
ppltrst, pplfair, pplhlp	Social trust scale (Breyer, 2015): Most people can be trusted or you can’t be too careful, Most people try to take advantage of you, or try to be fair, Most of the time people helpful or mostly looking out for themselves	From 0 = Low trust to 10 = High trust
sclmeet	“How often do you meet socially with friends, relatives or work colleagues?”	From 1 = Never to 7 = Every day
sclact	“Compared to other people of your age, how often would you say you take part in social activities?”	From 1 = Much less than most to 5 = Much more than most
Lifestyle behaviours (for ESS round 7 only)		
dosprt	“On how many of the last 7 days did you walk quickly, do sports or other physical activity for 30 minutes or longer?”	Number of days
*cgtsday*	“How many cigarettes do you smoke on a typical day?”	Number of cigarettes
*cgtssmke*	Cigarettes smoking behaviour	From 1 = I smoke daily to 5 = I have never smoked
cigarettes	Smoking cigarettes (computed based on ‘cgtsday’ and ‘cgtssmke’)	Number of cigarettes
alcfreq	“In the last 12 months, that is since [MONTH, YEAR], how often have you had a drink containing alcohol? This could be wine, beer, cider, spirits or other drinks containing alcohol.”	From 1 = Every day to 7 = Never
alcbnge	“This card shows six different examples of how much alcohol a person might drink on a single occasion. […] In the last 12 months, how often have you drunk this amount of alcohol or more on a single occasion?”	From 1 = Daily or almost daily to 5 = Never
bdmsindex	Body mass index (computed based on ‘height’ and ‘weight’)	weight/(square of (height/100))
*height*	“What is your height without shoes?”	In cm
weight	“What is your weight without shoes?”	In kg
Well-being and psychological distress		
happy	“Taking all things together, how happy would you say you are?”	From 0 = Extremely unhappy to 10 = Extremely happy
health	“How is your health in general? Would you say it is …”	From 1 = Very good to 5 = Very bad
fltdpr	“Using this card, please tell me how much of the time during the past week you felt depressed?”	1 = None or almost none of the time, 2 = Some of the time, 3 = Most of the time, 4 = All or almost all of the time
flteeff	“Using this card, please tell me how much of the time during the past week you felt that everything you did was an effort?”	1 = None or almost none of the time, 2 = Some of the time, 3 = Most of the time, 4 = All or almost all of the time
slprl	“Using this card, please tell me how much of the time during the past week your sleep was restless?”	1 = None or almost none of the time, 2 = Some of the time, 3 = Most of the time, 4 = All or almost all of the time
enjlf	“Using this card, please tell me how much of the time during the past week you enjoyed life?”	1 = None or almost none of the time, 2 = Some of the time, 3 = Most of the time, 4 = All or almost all of the time
fltsd	“Using this card, please tell me how much of the time during the past week you felt sad?”	1 = None or almost none of the time, 2 = Some of the time, 3 = Most of the time, 4 = All or almost all of the time
cldgng	“Using this card, please tell me how much of the time during the past week you could not get going? … In the sense of ‘felt lethargic and lacked motivation’.”	1 = None or almost none of the time, 2 = Some of the time, 3 = Most of the time, 4 = All or almost all of the time
