# Supplementary material for: Trends in loneliness in 17 European countries between 2006 and 2015: A secondary analysis of data from the European Social Survey
Source: J Health Psychol. 2024 Sep 18;30(7):1680–97. doi: 10.1177/13591053241278473 (PMC12166141; doi:10.1177/13591053241278473)
Supplement: sj-docx-1-hpq-10.1177_13591053241278473 – Supplemental material for Trends in loneliness in 17 European countries between 2006 and 2015: A secondary analysis of data from the European Social Survey [file sj-docx-1-hpq-10.1177_13591053241278473.docx]

**Table S1. Weighted descriptive characteristics of the sample across rounds 3, 5, 6, and 7 of the European Social Survey.**

| **Subgroup** | **Prevalence % (SE)** |
| --- | --- |
| **ESS round** |  |
| 3 (2006-07) | 25.12 (0.19) |
| 5 (2010-12) | 24.74 (0.19) |
| 6 (2012-13) | 24.96 (0.19) |
| 7 (2014-15) | 25.19 (0.19) |
| **Loneliness** *Mean (SE)* | 1.38 (0.00) |
| **Loneliness frequency** (last week) |  |
| None or almost none | 71.86 (0.20) |
| Some | 21.08 (0.18) |
| Most | 4.69 (0.09) |
| All or almost all | 2.37 (0.06) |
| ***Sociodemographic*** |  |
| **Sex** |  |
| Male | 48.45 (0.22) |
| Female | 51.55 (0.22) |
| **Age group** |  |
| 15-29 years | 21.99 (0.19) |
| 30-49 years | 33.64 (0.20) |
| 50-64 years | 24.13 (0.18) |
| 65-79 years | 15.70 (0.15) |
| >79 years | 4.55 (0.09) |
| **European region** |  |
| Northern | 6.84 (0.02) |
| Southern | 14.61 (0.05) |
| Western | 65.03 (0.08) |
| Central and Eastern | 13.51 (0.04) |
| **Country** |  |
| Belgium | 27.98 (0.01) |
| Switzerland | 20.52 (0.01) |
| Germany | 21.84 (0.09) |
| Denmark | 1.41 (0.01) |
| Estonia | 0.35 (0.00) |
| Spain | 11.99 (0.04) |
| Finland | 1.38 (0.00) |
| France | 16.17 (0.11) |
| United Kingdom | 16.85 (0.10) |
| Hungary | 2.67 (0.01) |
| Ireland | 1.09 (0.01) |
| Netherlands | 4.24 (0.03) |
| Norway | 1.23 (0.01) |
| Poland | 10.31 (0.03) |
| Portugal | 2.62 (0.02) |
| Sweden | 2.48 (0.01) |
| Slovenia | 0.54 (0.00) |
| **Living in a city** | 64.14 (0.20) |
| **Living alone** | 15.19 (0.14) |
| **Disability** | 3.43 (0.08) |
| **Immigration status** |  |
| No immigrant background | 88.32 (0.16) |
| Second generation | 2.79 (0.08) |
| First generation | 8.89 (0.14) |
| **Not in employment/education** | 38.59 (0.21) |
| **Level of education** |  |
| Low | 36.05 (0.22) |
| Intermediate | 46.01 (0.22) |
| High | 17.94 (0.16) |
| **Not religious** | 41.25 (0.21) |
| **Severe social isolation** |  |
| Total | 3.26 (0.04) |
| Working-age population (15-64 years) | 1.73 (0.04) |
| > 64 years | 9.33 (0.27) |
| ***Social factors*** |  |
| Victimization | 18.46 (0.17) |
| Emotional support | 94.41 (0.10) |
| Social trust *Mean (SE)* | 5.20 (0.01) |
| Social meetings *Mean (SE)* | 4.96 (0.01) |
| Social activities *Mean (SE)* | 2.75 (0.00) |
| ***Well-being and psychological distress ^a^*** |  |
| Happiness *Mean (SE)* | 7.43 (0.01) |
| General health *Mean (SE)* | 2.23 (0.00) |
| Enjoying life *Mean (SE)* | 2.94 (0.00) |
| Symptoms of depression *Mean (SE)* | 1.48 (0.00) |
| Feeling everything like effort *Mean (SE)* | 1.71 (0.00) |
| Restless sleep *Mean (SE)* | 1.80 (0.01) |
| Sadness *Mean (SE)* | 1.54 (0.00) |
| Lethargy and lack of motivation *Mean (SE)* | 1.54 (0.00) |
| ***Lifestyle behaviours ^b^*** |  |
| Physical activity *Mean (SE)* | 3.14 (0.02) |
| Cigarettes smoked daily *Mean (SE)* | 3.22 (0.06) |
| Alcohol use *Mean (SE)* | 4.19 (0.02) |
| Binge drinking *Mean (SE)* | 3.84 (0.01) |
| Body mass index *Mean (SE)* | 25.62 (0.05) |

*se:* standard error, *ESS:* European Social Survey.

*^a^:* data not collected at ESS round 5 except for happiness and general health.

*^b^:* data collected at ESS round 7 only.
