# Supplementary material for: Trends in loneliness in 17 European countries between 2006 and 2015: A secondary analysis of data from the European Social Survey
Source: J Health Psychol. 2024 Sep 18;30(7):1680–97. doi: 10.1177/13591053241278473 (PMC12166141; doi:10.1177/13591053241278473)
Supplement: sj-docx-2-hpq-10.1177_13591053241278473 – Supplemental material for Trends in loneliness in 17 European countries between 2006 and 2015: A secondary analysis of data from the European Social Survey [file sj-docx-2-hpq-10.1177_13591053241278473.docx]

**Table S2. Loneliness weighted prevalence (and standard error) according to round 3, 5, 6 and 7 of the European Social Survey (ESS).**

| **Loneliness frequency** | **ESS round**  **3 (2006-07)** | **ESS round**  **5 (2010-12)** | **ESS round**  **6 (2012-13)** | **ESS round**  **7 (2014-15)** |
| --- | --- | --- | --- | --- |
| None or almost none of the time | 70.12 (0.39) | 72.21 (0.40) | 71.88 (0.39) | 73.22 (0.39) |
| Some of the time | 22.42 (0.36) | 21.03 (0.37) | 20.90 (0.35) | 19.97 (0.35) |
| Most of the time | 4.88 (0.19) | 4.94 (0.20) | 4.59 (0.18) | 4.37 (0.18) |
| All or almost all of the time | 2.58 (0.13) | 1.83 (0.11) | 2.64 (0.14) | 2.43 (0.14) |
