# Supplementary material for: Trends in loneliness in 17 European countries between 2006 and 2015: A secondary analysis of data from the European Social Survey
Source: J Health Psychol. 2024 Sep 18;30(7):1680–97. doi: 10.1177/13591053241278473 (PMC12166141; doi:10.1177/13591053241278473)
Supplement: sj-docx-3-hpq-10.1177_13591053241278473 – Supplemental material for Trends in loneliness in 17 European countries between 2006 and 2015: A secondary analysis of data from the European Social Survey [file sj-docx-3-hpq-10.1177_13591053241278473.docx]

**Table S3. Results of weighted regression analysis predicting loneliness by the independent variable of interest and controlling for the round of the European Social Survey (not shown).**

| **Independent variable** | **estimate (se)** | ***t*** | ***p*** |
| --- | --- | --- | --- |
| ESS round 3 | Reference |  |  |
| ESS round 5 | -0.035 (0.008) | -4.258 | < 0.001 |
| ESS round 6 | -0.019 (0.008) | -2.299 | 0.022 |
| ESS round 7 | -0.039 (0.008) | -4.646 | < 0.001 |
| Female | 0.110 (0.006) | 18.711 | < 0.001 |
| 15-29 years | Reference |  |  |
| 30-49 years | -0.019 (0.008) | -2.388 | 0.017 |
| 50-64 years | 0.009 (0.009) | 1.031 | 0.303 |
| 65-79 years | 0.047 (0.010) | 4.745 | <0.001 |
| >79 years | 0.305 (0.019) | 16.233 | <0.001 |
| Northern  Europe | Reference |  |  |
| Southern | 0.146 (0.008) | 18.409 | < 0.001 |
| Western | 0.074 (0.005) | 13.757 | < 0.001 |
| Central and Eastern | 0.174 (0.008) | 20.563 | < 0.001 |
| Living in a city | 0.031 (0.006) | 5.069 | < 0.001 |
| Living alone | 0.489 (0.010) | 51.413 | < 0.001 |
| Presence of disability | 0.373 (0.022) | 17.299 | < 0.001 |
| No immigrant background | Reference |  |  |
| Second generation | 0.038 (0.021) | 1.827 | 0.068 |
| First generation | 0.105 (0.012) | 8.672 | < 0.001 |
| Not in employment/education | 0.188 (0.006) | 29.585 | < 0.001 |
| Low education | Reference |  |  |
| Intermediate | -0.135 (0.007) | -18.416 | < 0.001 |
| High | -0.195 (0.008) | -23.513 | < 0.001 |
| Not religious | -0.009 (0.006) | -1.537 | 0.124 |
| Severe social isolation | 0.482 (0.022) | 21.693 | < 0.001 |

*se:* standard error.
