# Supplementary material for: Trends in loneliness in 17 European countries between 2006 and 2015: A secondary analysis of data from the European Social Survey
Source: J Health Psychol. 2024 Sep 18;30(7):1680–97. doi: 10.1177/13591053241278473 (PMC12166141; doi:10.1177/13591053241278473)
Supplement: sj-pdf-4-hpq-10.1177_13591053241278473 – Supplemental material for Trends in loneliness in 17 European countries between 2006 and 2015: A secondary analysis of data from the European Social Survey [file sj-pdf-4-hpq-10.1177_13591053241278473.pdf]

Figure S2. Trends in weighted prevalence of loneliness category - based on time during the past week that respondents felt lonely - according to the following sociodemographic characteristics: A) sex, B) age group, C) immigrant status, D) European region, E) country, F) city, G) living alone, H) disability, I) employment/education, L) level of education, M) religiosity, and N) severe social isolation.

A

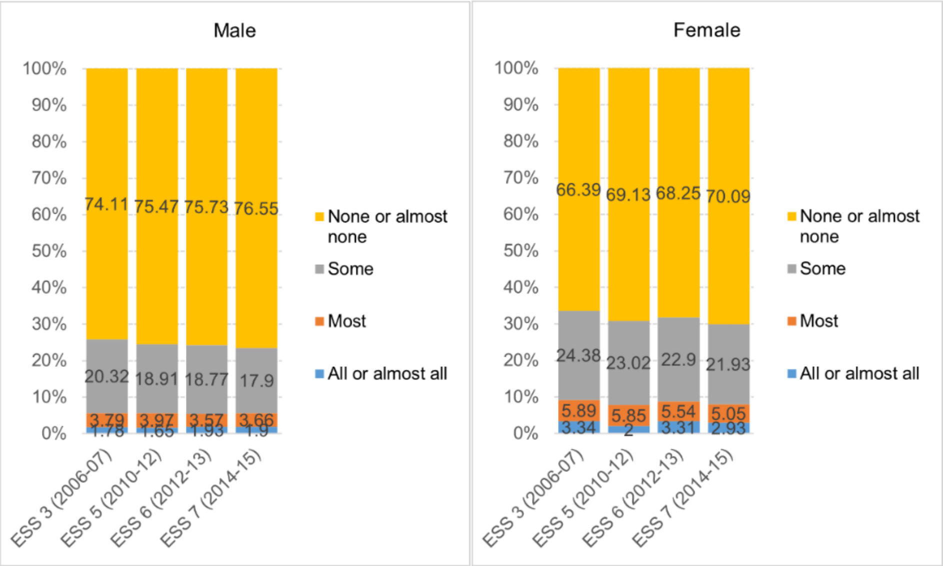

B

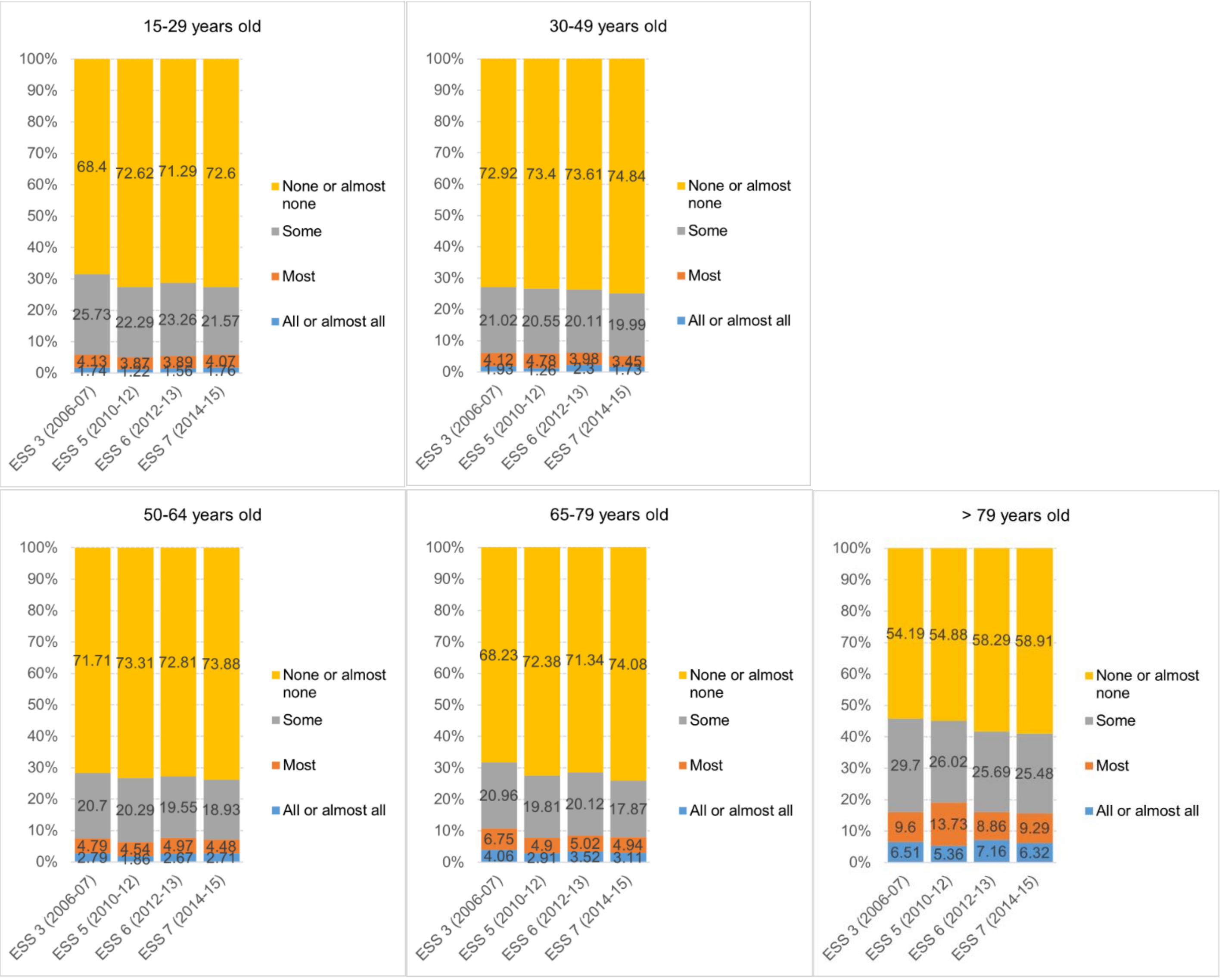

C

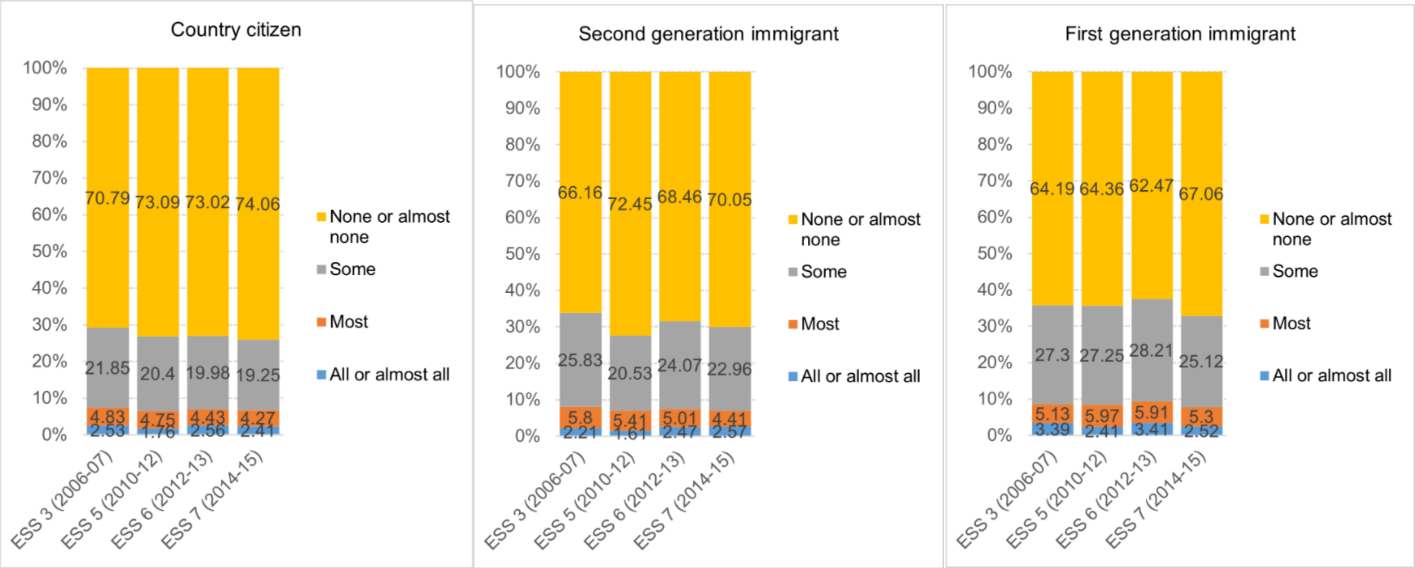
Country citizen
Second generation immigrant
First generation immigrant
100%
90%
80%
70%
60%
50%
40%
30%
20%
10%
0%
70.79 73.09 73.02 74.06
21.85 20.4 19.98 19.25
4.83 4.75 4.43 4.27
66.16 72.45 68.46 70.05
25.83 20.53 24.07 22.96
5.8 5.41 5.01 4.41
2.21 2.47 2.57
64.19 64.36 62.47 67.06
27.3 27.25 28.21 25.12
5.13 5.97 5.91 5.3
3.39 2.41 3.41 2.52
None or almost none
Some
Most
All or almost all
ESS 3 (2006-07)
ESS 5 (2010-12)
ESS 6 (2012-13)
ESS 7 (2014-15)

D

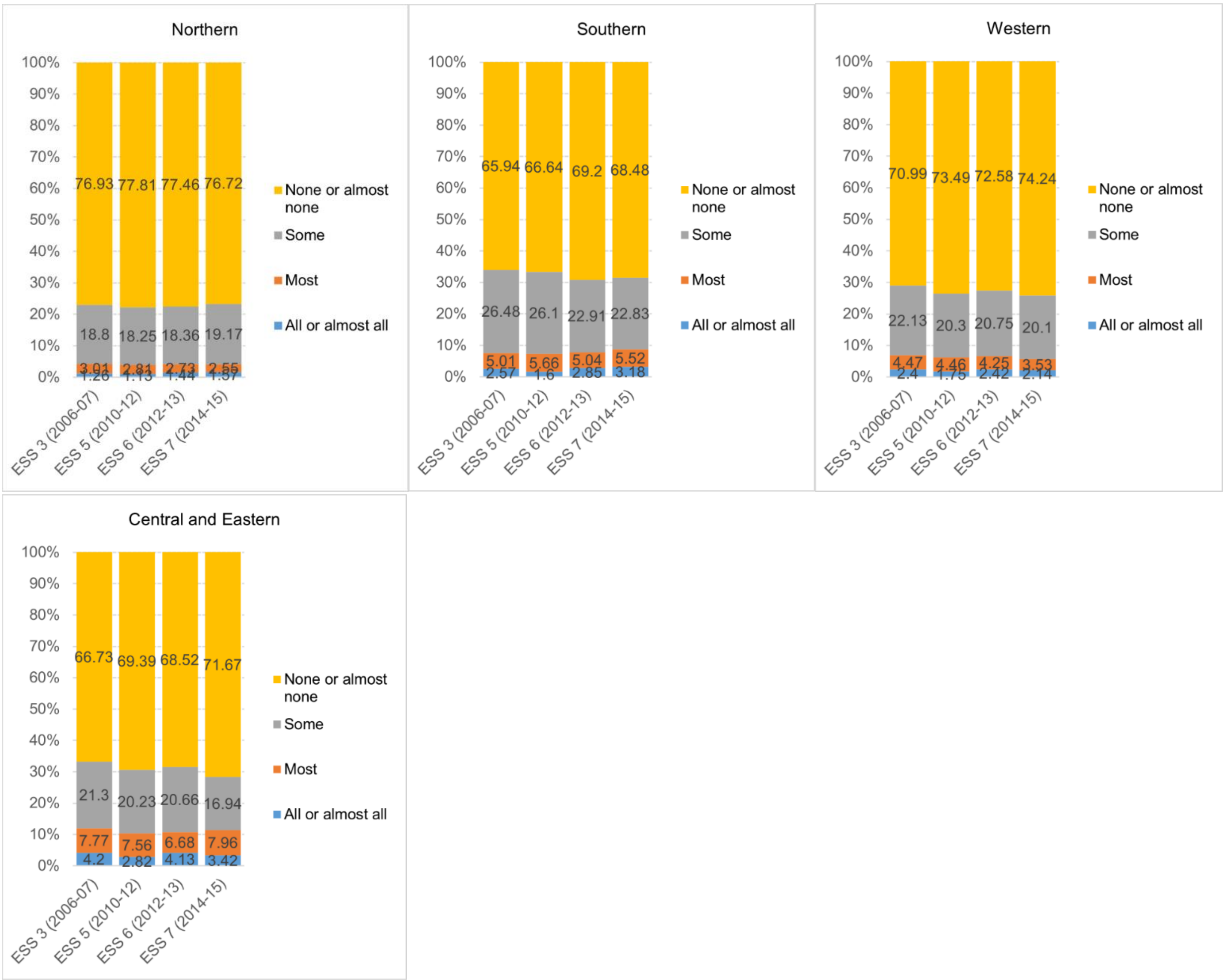
Northern
Southern
Western
Central and Eastern
100%
90%
80%
70%
60%
50%
40%
30%
20%
10%
0%
76.93 77.81 77.46 76.72
18.8 18.25 18.36 19.17
2.81 2.73 2.55
65.94 66.64 69.2 68.48
26.48 26.1 22.91 22.83
5.01 5.66 5.04 5.52
2.57 1.6 2.85 3.18
70.99 73.49 72.58 74.24
22.13 20.3 20.75 20.1
4.47 4.46 4.25 3.53
2.4 1.75 2.42 2.14
66.73 69.39 68.52 71.67
21.3 20.23 20.66 16.94
7.77 7.56 6.68 7.96
4.2 2.82 4.13 3.42
None or almost none
Some
Most
All or almost all
ESS 3 (2006-07)
ESS 5 (2010-12)
ESS 6 (2012-13)
ESS 7 (2014-15)

E

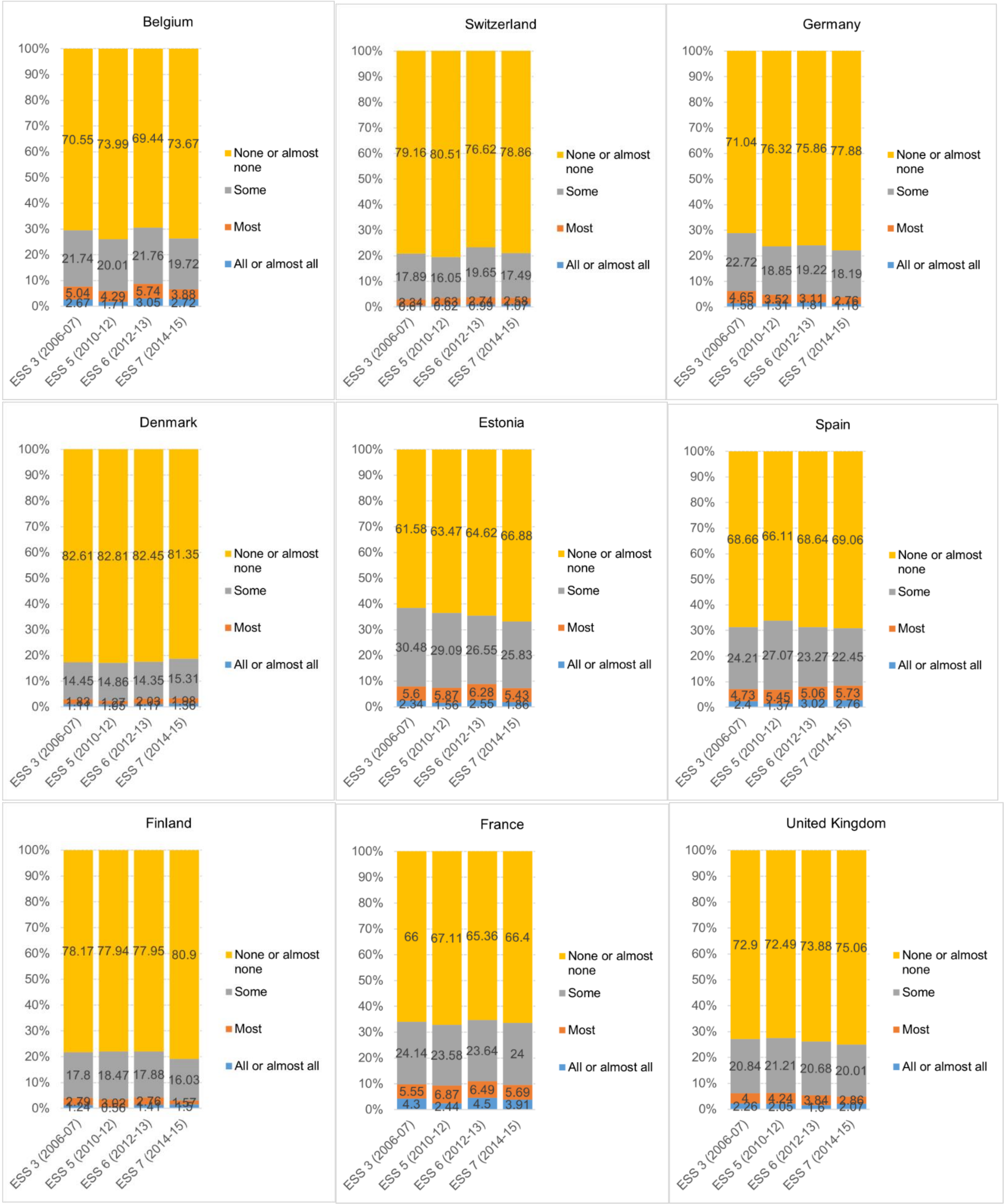
Belgium
70.55
73.99
69.44
73.67
21.74
20.01
21.76
19.72
5.04
4.29
5.74
3.88
2.67
3.05
2.72
Switzerland
79.16
80.51
76.62
78.86
17.89
16.05
19.65
17.49
Germany
71.04
76.32
75.86
77.88
22.72
18.85
19.22
18.19
4.65
3.52
3.11
2.76
Denmark
82.61
82.81
82.45
81.35
14.45
14.86
14.35
15.31
Estonia
61.58
63.47
64.62
66.88
30.48
29.09
26.55
25.83
5.6
5.87
6.28
5.43
2.34
2.55
Spain
68.66
66.11
68.64
69.06
24.21
27.07
23.27
22.45
4.73
5.45
5.06
5.73
3.02
2.76
Finland
78.17
77.94
77.95
80.9
17.8
18.47
17.88
16.03
France
66
67.11
65.36
66.4
24.14
23.58
23.64
24
5.55
6.87
6.49
5.69
4.3
2.44
4.5
3.91
United Kingdom
72.9
72.49
73.88
75.06
20.84
21.21
20.68
20.01
4
4.24
3.84
2.86
2.26
2.05
2.07
100%
90%
80%
70%
60%
50%
40%
30%
20%
10%
0%
ESS 3 (2006-07)
ESS 5 (2010-12)
ESS 6 (2012-13)
ESS 7 (2014-15)
None or almost none
Some
Most
All or almost all

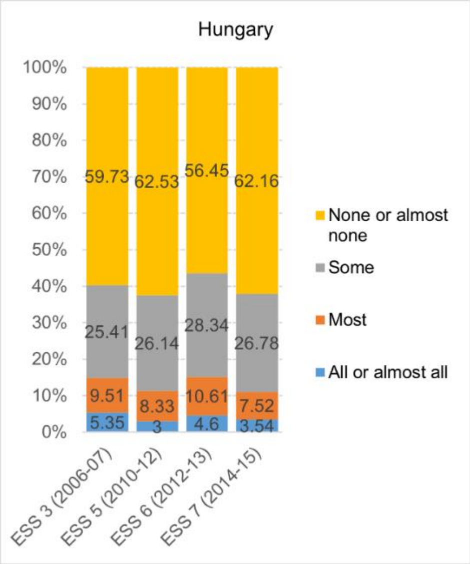
Hungary
100%
90%
80%
70%
60%
50%
40%
30%
20%
10%
0%
59.73
62.53
56.45
62.16
25.41
26.14
28.34
26.78
9.51
8.33
10.61
7.52
5.35
3
4.6
3.54
None or almost none
Some
Most
All or almost all
ESS 3 (2006-07)
ESS 5 (2010-12)
ESS 6 (2012-13)
ESS 7 (2014-15)

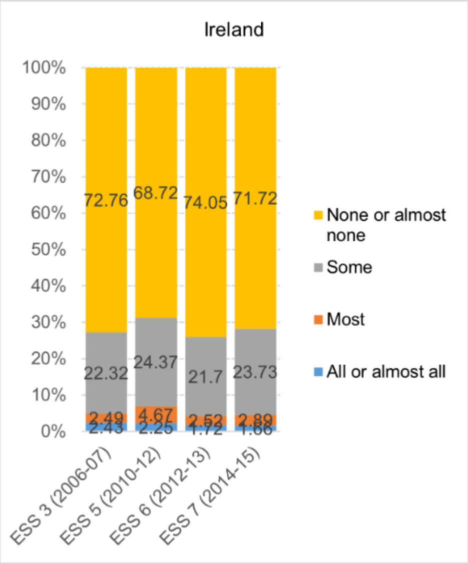
Ireland
100%
90%
80%
70%
60%
50%
40%
30%
20%
10%
0%
72.76
68.72
74.05
71.72
22.32
24.37
21.7
23.73
2.49
4.67
2.52
2.88
2.43
2.25
1.72
1.66
None or almost none
Some
Most
All or almost all
ESS 3 (2006-07)
ESS 5 (2010-12)
ESS 6 (2012-13)
ESS 7 (2014-15)

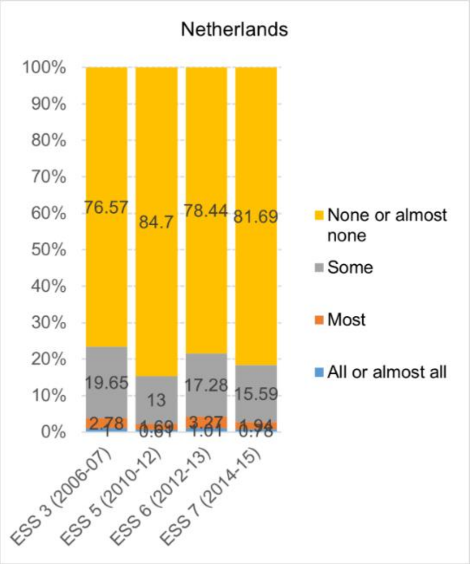
Netherlands
100%
90%
80%
70%
60%
50%
40%
30%
20%
10%
0%
76.57
84.7
78.44
81.69
19.65
13
17.28
15.59
2.78
1.69
3.27
1.94
1
0.61
1.01
0.78
None or almost none
Some
Most
All or almost all
ESS 3 (2006-07)
ESS 5 (2010-12)
ESS 6 (2012-13)
ESS 7 (2014-15)

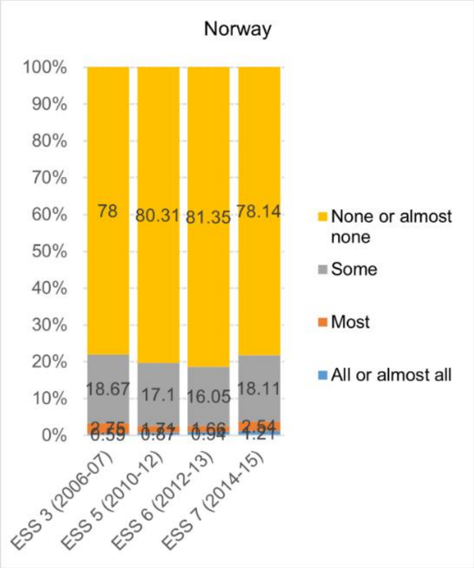
Norway
100%
90%
80%
70%
60%
50%
40%
30%
20%
10%
0%
78
80.31
81.35
78.14
18.67
17.1
16.05
18.11
2.75
1.71
1.66
2.54
0.59
0.87
0.94
1.21
None or almost none
Some
Most
All or almost all
ESS 3 (2006-07)
ESS 5 (2010-12)
ESS 6 (2012-13)
ESS 7 (2014-15)

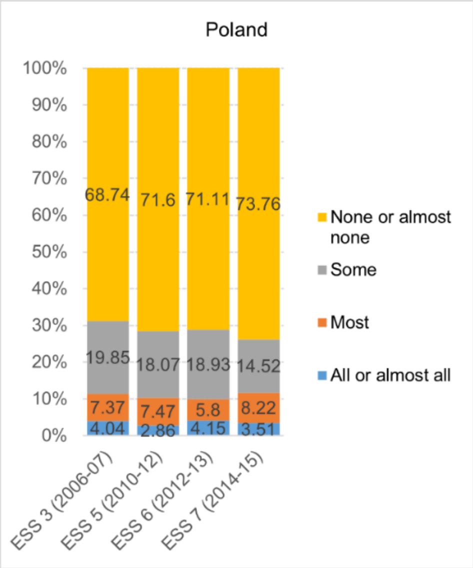
Poland
100%
90%
80%
70%
60%
50%
40%
30%
20%
10%
0%
68.74
71.6
71.11
73.76
19.85
18.07
18.93
14.52
7.37
7.47
5.8
8.22
4.04
2.86
4.15
3.51
None or almost none
Some
Most
All or almost all
ESS 3 (2006-07)
ESS 5 (2010-12)
ESS 6 (2012-13)
ESS 7 (2014-15)

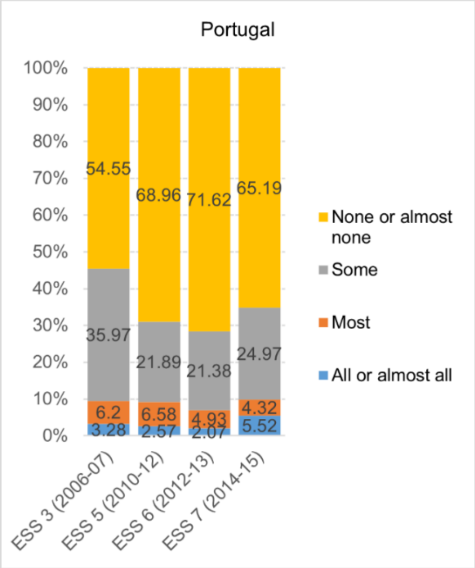
Portugal
100%
90%
80%
70%
60%
50%
40%
30%
20%
10%
0%
54.55
68.96
71.62
65.19
35.97
21.89
21.38
24.97
6.2
6.58
4.93
4.32
3.28
2.57
2.07
5.52
None or almost none
Some
Most
All or almost all
ESS 3 (2006-07)
ESS 5 (2010-12)
ESS 6 (2012-13)
ESS 7 (2014-15)

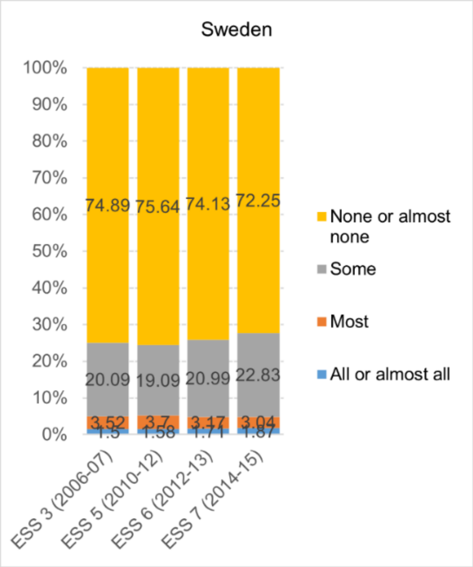
Sweden
100%
90%
80%
70%
60%
50%
40%
30%
20%
10%
0%
74.89
75.64
74.13
72.25
20.09
19.09
20.99
22.83
3.52
3.7
3.17
3.04
1.5
1.58
1.71
1.87
None or almost none
Some
Most
All or almost all
ESS 3 (2006-07)
ESS 5 (2010-12)
ESS 6 (2012-13)
ESS 7 (2014-15)

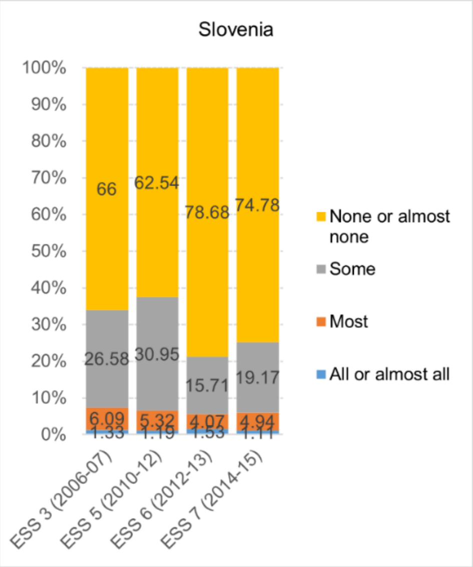
Slovenia
100%
90%
80%
70%
60%
50%
40%
30%
20%
10%
0%
66
62.54
78.68
74.78
26.58
30.95
15.71
19.17
6.09
5.32
4.07
4.94
1.33
1.19
1.53
1.11
None or almost none
Some
Most
All or almost all
ESS 3 (2006-07)
ESS 5 (2010-12)
ESS 6 (2012-13)
ESS 7 (2014-15)

F

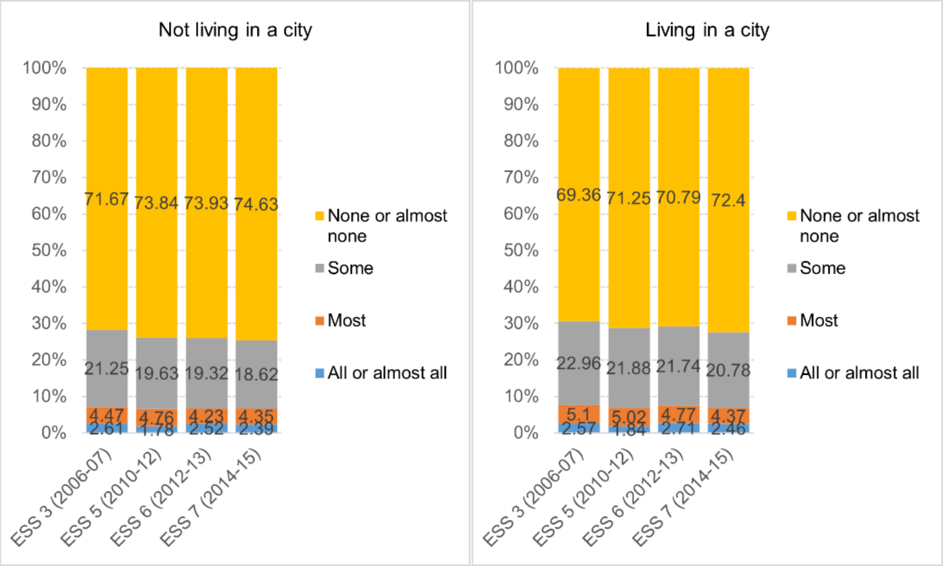
Not living in a city
Living in a city
100%
90%
80%
70%
60%
50%
40%
30%
20%
10%
0%
71.67 73.84 73.93 74.63
21.25 19.63 19.32 18.62
4.47 4.76 4.23 4.35
2.61 1.78 2.52 2.39
69.36 71.25 70.79 72.4
22.96 21.88 21.74 20.78
5.1 5.02 4.77 4.37
2.57 1.84 2.71 2.46
None or almost none
Some
Most
All or almost all
ESS 3 (2006-07)
ESS 5 (2010-12)
ESS 6 (2012-13)
ESS 7 (2014-15)

G

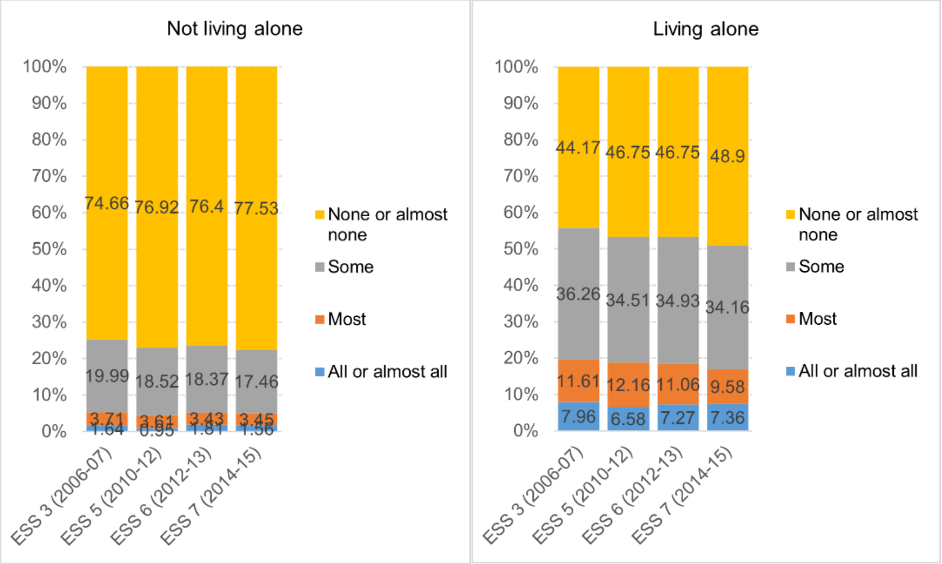
Not living alone
Living alone
100%
90%
80%
70%
60%
50%
40%
30%
20%
10%
0%
74.66 76.92 76.4 77.53
19.99 18.52 18.37 17.46
3.71 3.61 3.43 3.45
1.64 0.95 1.81 1.56
44.17 46.75 46.75 48.9
36.26 34.51 34.93 34.16
11.61 12.16 11.06 9.58
7.96 6.58 7.27 7.36
None or almost none
Some
Most
All or almost all
ESS 3 (2006-07)
ESS 5 (2010-12)
ESS 6 (2012-13)
ESS 7 (2014-15)

H

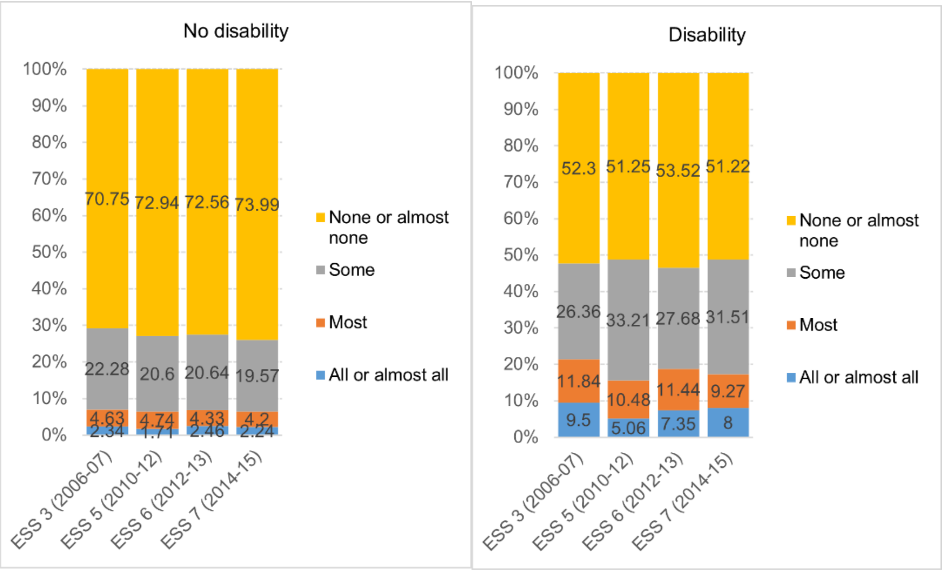
No disability
Disability
100%
90%
80%
70%
60%
50%
40%
30%
20%
10%
0%
70.75 72.94 72.56 73.99
22.28 20.6 20.64 19.57
4.63 4.74 4.33 4.2
2.34 1.71 2.46 2.24
52.3 51.25 53.52 51.22
26.36 33.21 27.68 31.51
11.84 10.48 11.44 9.27
9.5 5.06 7.35 8
None or almost none
Some
Most
All or almost all
ESS 3 (2006-07)
ESS 5 (2010-12)
ESS 6 (2012-13)
ESS 7 (2014-15)

I

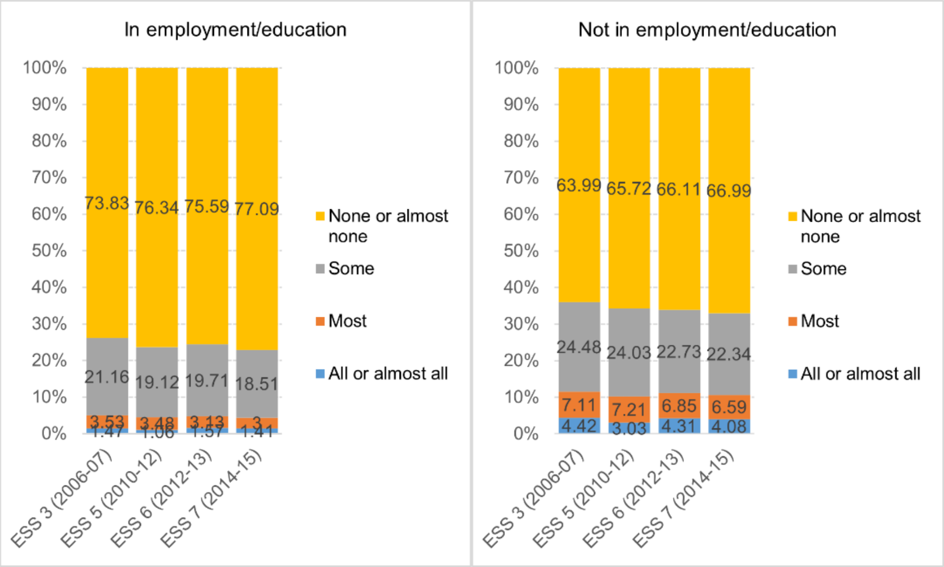
In employment/education
100%
90%
80%
70%
60%
50%
40%
30%
20%
10%
0%
73.83
76.34
75.59
77.09
21.16
19.12
19.71
18.51
3.53
3.48
3.13
3
1.47
1.06
1.57
1.41
ESS 3 (2006-07)
ESS 5 (2010-12)
ESS 6 (2012-13)
ESS 7 (2014-15)
None or almost none
Some
Most
All or almost all
Not in employment/education
63.99
65.72
66.11
66.99
24.48
24.03
22.73
22.34
7.11
7.21
6.85
6.59
4.42
3.03
4.31
4.08

L

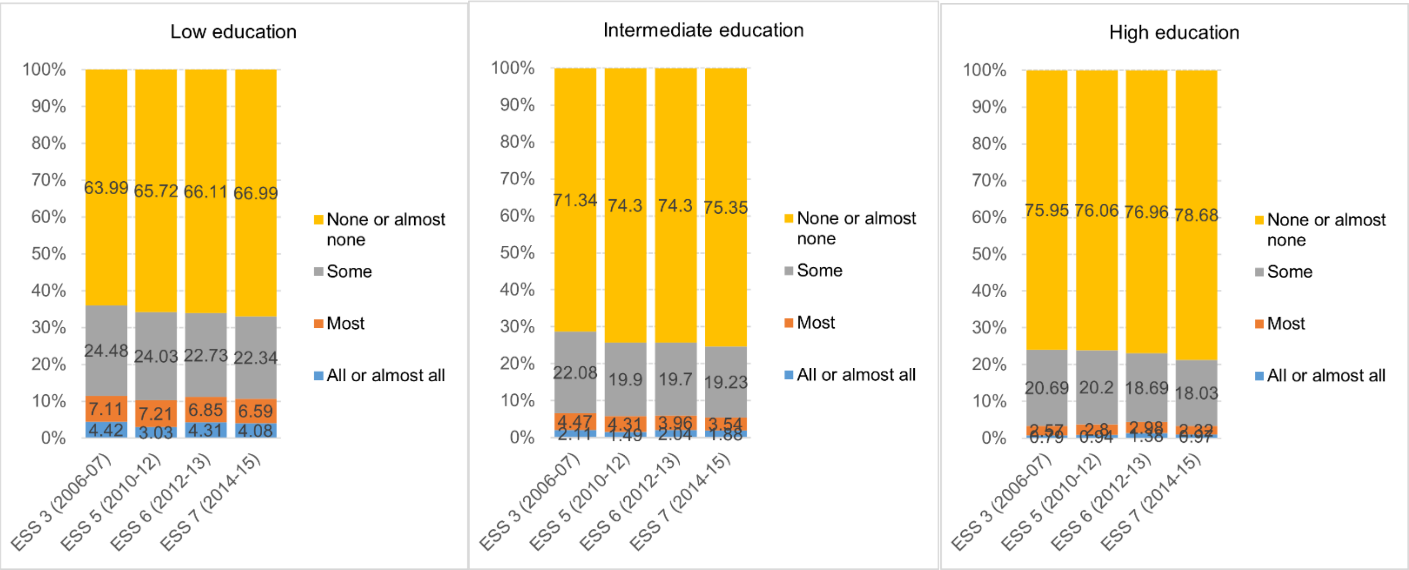
Low education
63.99
65.72
66.11
66.99
24.48
24.03
22.73
22.34
7.11
7.21
6.85
6.59
4.42
3.03
4.31
4.08
Intermediate education
71.34
74.3
74.3
75.35
22.08
19.9
19.7
19.23
4.47
4.31
3.96
3.54
2.11
1.49
2.04
1.88
High education
75.95
76.06
76.96
78.68
20.69
20.2
18.69
18.03
2.57
2.8
2.98
2.32
0.79
0.94
1.38
0.97
ESS 3 (2006-07)
ESS 5 (2010-12)
ESS 6 (2012-13)
ESS 7 (2014-15)
None or almost none
Some
Most
All or almost all

M

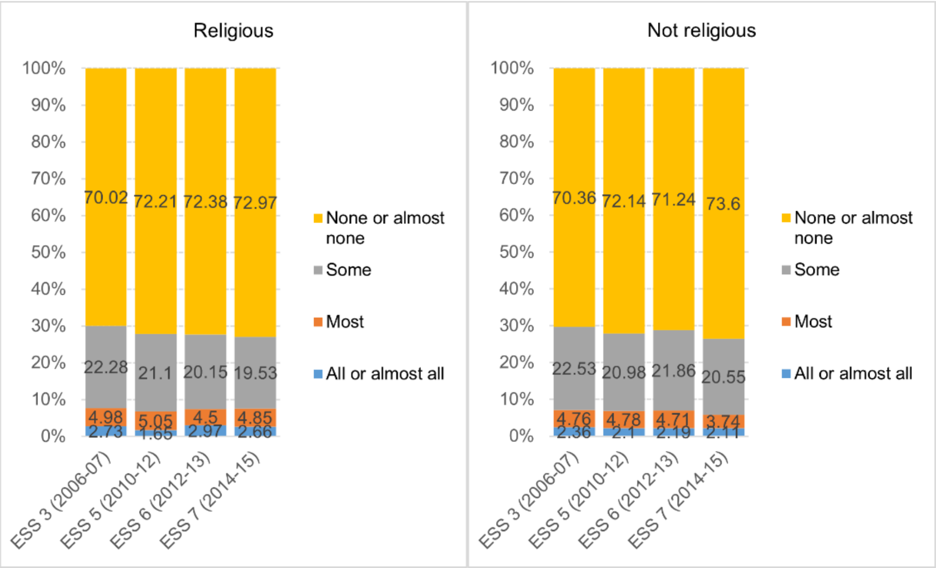
Religious
70.02
72.21
72.38
72.97
22.28
21.1
20.15
19.53
4.98
5.05
4.5
4.85
2.73
1.65
2.97
2.66
Not religious
70.36
72.14
71.24
73.6
22.53
20.98
21.86
20.55
4.76
4.78
4.71
3.74
2.36
2.1
2.19
2.11
ESS 3 (2006-07)
ESS 5 (2010-12)
ESS 6 (2012-13)
ESS 7 (2014-15)
None or almost none
Some
Most
All or almost all

N

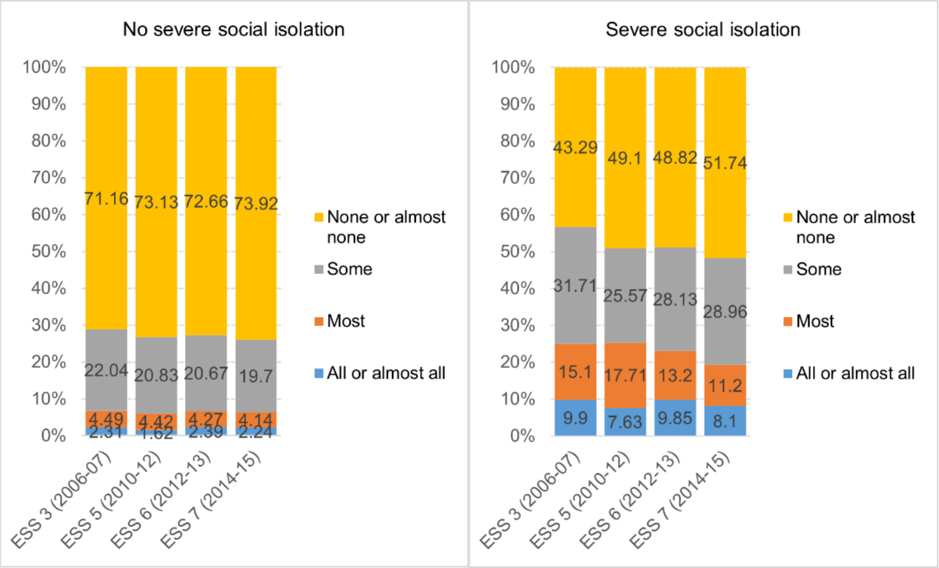
No severe social isolation
Severe social isolation
100%
90%
80%
70%
60%
50%
40%
30%
20%
10%
0%
71.16
73.13
72.66
73.92
22.04
20.83
20.67
19.7
4.49
4.42
4.27
4.14
2.31
1.62
2.39
2.24
43.29
49.1
48.82
51.74
31.71
25.57
28.13
28.96
15.1
17.71
13.2
11.2
9.9
7.63
9.85
8.1
None or almost none
Some
Most
All or almost all
ESS 3 (2006-07)
ESS 5 (2010-12)
ESS 6 (2012-13)
ESS 7 (2014-15)
