# Supplementary material for: Trends in loneliness in 17 European countries between 2006 and 2015: A secondary analysis of data from the European Social Survey
Source: J Health Psychol. 2024 Sep 18;30(7):1680–97. doi: 10.1177/13591053241278473 (PMC12166141; doi:10.1177/13591053241278473)
Supplement: sj-pdf-5-hpq-10.1177_13591053241278473 – Supplemental material for Trends in loneliness in 17 European countries between 2006 and 2015: A secondary analysis of data from the European Social Survey [file sj-pdf-5-hpq-10.1177_13591053241278473.pdf]

Figure S1. Trends in weighted mean of loneliness single-item measure according to the following sociodemographic characteristics: A) sex, B) age group, C) immigrant status, D) European region, E) country, F) city, G) living alone, H) disability, I) employment/education, L) level of education, M) religiosity, and N) severe social isolation.

A

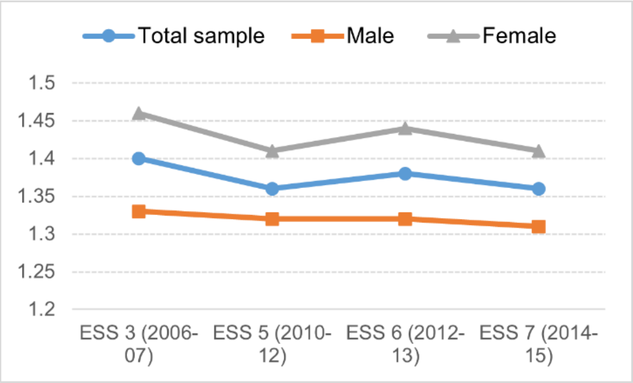

B

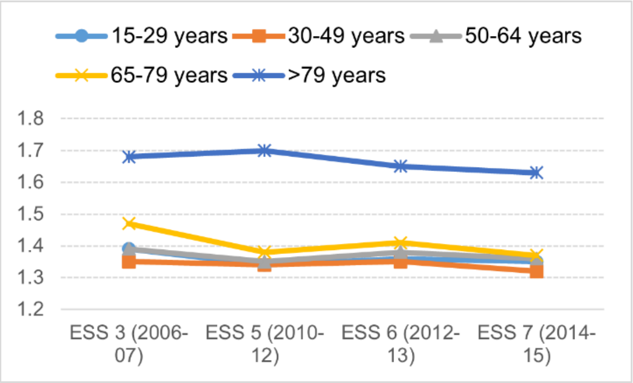

C

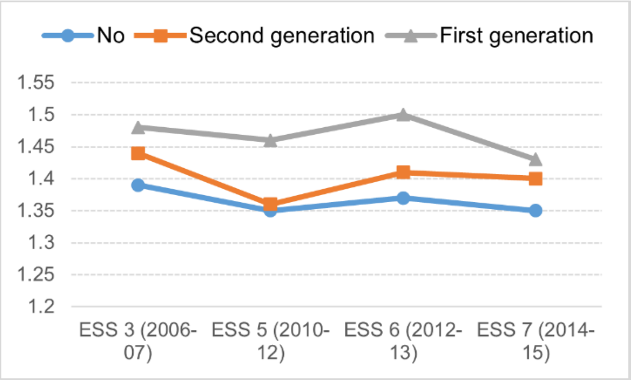

D

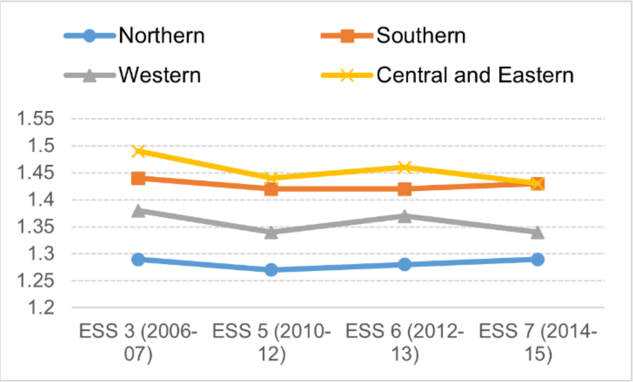

E

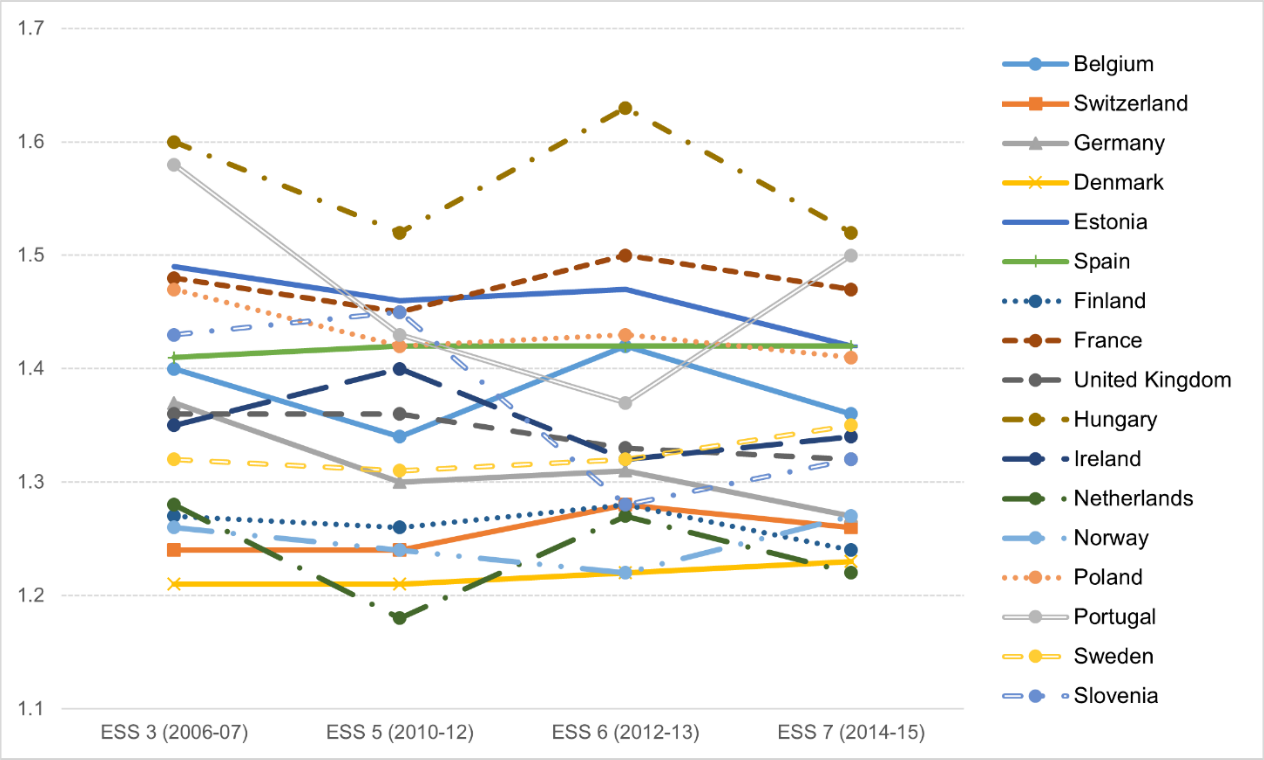

F

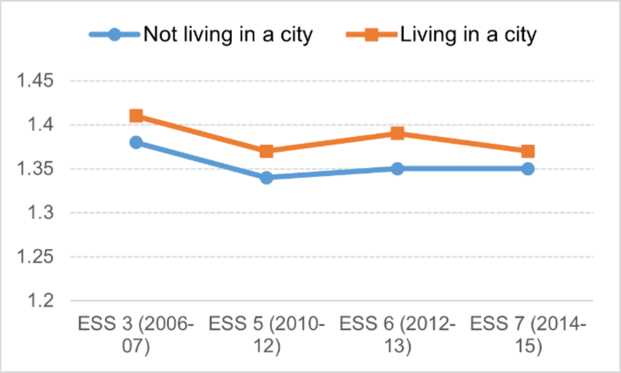
Not living in a city
Living in a city
1.45
1.4
1.35
1.3
1.25
1.2
ESS 3 (2006-07)
ESS 5 (2010-12)
ESS 6 (2012-13)
ESS 7 (2014-15)

G

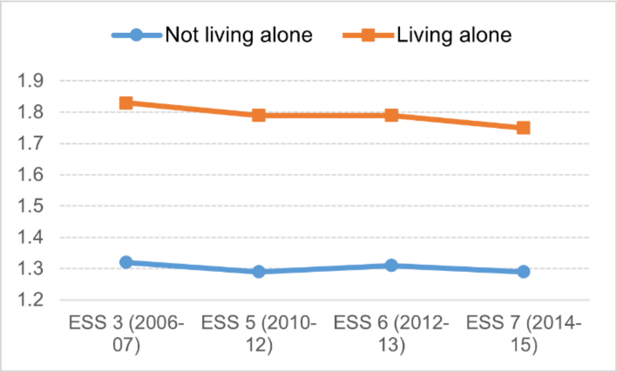
Not living alone
Living alone
1.9
1.8
1.7
1.6
1.5
1.4
1.3
1.2
ESS 3 (2006-07)
ESS 5 (2010-12)
ESS 6 (2012-13)
ESS 7 (2014-15)

H

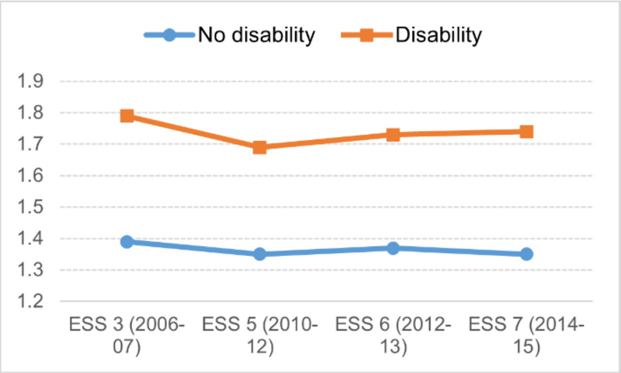
No disability
Disability
1.9
1.8
1.7
1.6
1.5
1.4
1.3
1.2
ESS 3 (2006-07)
ESS 5 (2010-12)
ESS 6 (2012-13)
ESS 7 (2014-15)

I

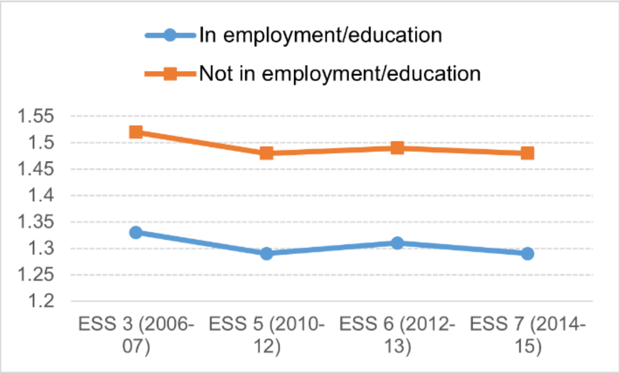
In employment/education
Not in employment/education
1.55
1.5
1.45
1.4
1.35
1.3
1.25
1.2
ESS 3 (2006-07)
ESS 5 (2010-12)
ESS 6 (2012-13)
ESS 7 (2014-15)

L

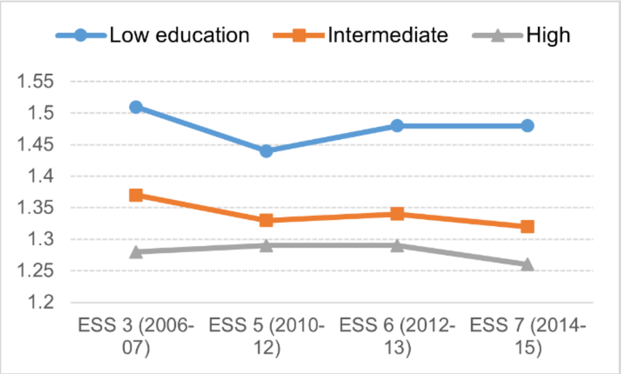
Low education
Intermediate
High
1.55
1.5
1.45
1.4
1.35
1.3
1.25
1.2
ESS 3 (2006-07)
ESS 5 (2010-12)
ESS 6 (2012-13)
ESS 7 (2014-15)

M

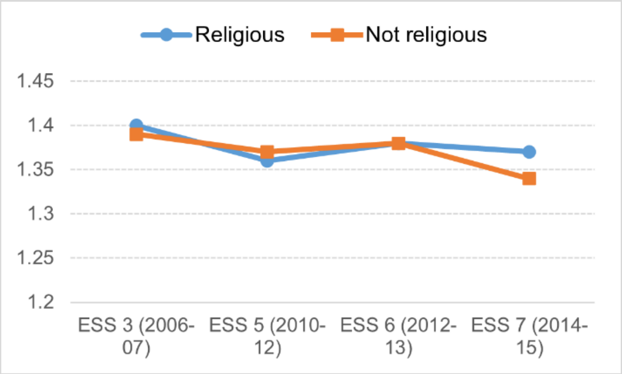
Religious
Not religious
1.45
1.4
1.35
1.3
1.25
1.2
ESS 3 (2006-07)
ESS 5 (2010-12)
ESS 6 (2012-13)
ESS 7 (2014-15)

N

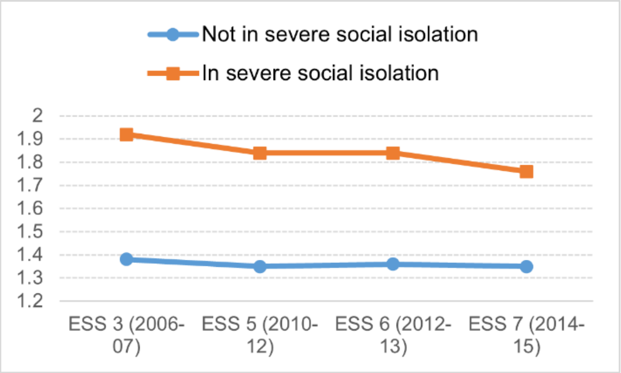
Not in severe social isolation
In severe social isolation
2
1.9
1.8
1.7
1.6
1.5
1.4
1.3
1.2
ESS 3 (2006-07)
ESS 5 (2010-12)
ESS 6 (2012-13)
ESS 7 (2014-15)
